# The wave nature of the action potential

**DOI:** 10.3389/fncel.2025.1467466

**Published:** 2025-04-25

**Authors:** Vitaly L. Galinsky, Lawrence R. Frank

**Affiliations:** ^1^Center for Scientific Computation in Imaging, University of California at San Diego, La Jolla, CA, United States; ^2^Center for Functional MRI, University of California at San Diego, La Jolla, CA, United States

**Keywords:** action potential, neuron, critical dynamics, wave dynamics, brain physics

## Abstract

An alternative to the standard Hodgkin-Huxley model for the action potential in axons is presented. It is based on our recently developed theory of electric field wave propagation in anisotropic and inhomogeneous brain tissues, which has been shown to explain a broad range of observed coherent synchronous brain electrical processes. We demonstrate that this theory also explains the spiking behavior of single neurons, thereby bridging the gap between the fundamental element of brain electrical activity—the neuron—and large-scale coherent synchronous electrical activity. We demonstrate that our recently developed theory of electric field wave propagation in anisotropic and inhomogeneous brain tissues, which has been shown to explain a broad range of observed coherent synchronous brain electrical processes, also applies to the spiking behavior of single neurons, thus bridging the gap between the fundamental element of brain electrical activity (the neuron) and large-scale coherent synchronous electrical activity. Our analysis indicates that a non-linear system with several small parameters can mathematically describe the membrane interface of the axonal cellular system. This enables the rigorous derivation of an accurate yet simpler non-linear model through the formal small-parameter expansion. The resulting action potential model exhibits a smooth, continuous transition from the linear wave oscillatory regime to the non-linear spiking regime, as well as a critical transition to a non-oscillatory regime. These transitions occur with changes in the criticality parameter and include several different bifurcation types, representative of the various experimentally detected neuron types. This new theory addresses the limitations of the Hodgkin-Huxley model, including its inability to explain extracellular spiking, efficient brain synchronization, saltatory conduction along myelinated axons, and various other observed coherent macroscopic brain electrical phenomena. We also demonstrate that our approach recovers the standard cable axon theory, utilizing the relatively simple assumptions of piece-wise homogeneity and isotropy. However, the diffusion process described by the cable equation is not capable of supporting action potential propagation across a wide range of experimentally reported axon parameters.

## 1 Introduction

The Hodgkin-Huxley model for axonal electrical signaling (Hodgkin and Huxley, 1952) is a cornerstone of modern neuroscience and serves as the basis for the development of a wide range of complex models of brain electrical communication. This model, and a host of variations (e.g., Fitzhugh, 1961; Nagumo et al., 1962; Morris and Lecar, 1981; Izhikevich, 2003), (hereafter collectively referred to as “HH”) is based on the postulate that axons possess multiple voltage-gated channels that open and close in synchrony thereby producing a coherent persistent electrical wave or “spike” traveling along the axon. To parameterize experimentally observed spikes in support of this view, HH developed a model described by a reaction-diffusion process, where the non-linear reactive part with multiple parameters (the so-called “point neuron” HH model) describes the opening and closing of voltage-gated channels and, hence, provides very flexible interface for fitting locally measured currents to a wide variety of experimental conditions. The propagation of these local voltage spikes is then obtained by adding a linear diffusive term from the so-called “cable equation” (Hodgkin et al., 1946). However, despite the general utility, and universal acceptance of both parts, either individually or in concert as the joint diffusive-reactive HH model, several incontrovertible facts suggest an incompatibility of the joint HH model with observed brain electrical activity, such as its inability to account for extracellular spiking, efficient brain synchronization, and saltatory conduction along myelinated axons, to name just a few.

In retrospect, this should not be surprising, as neither reactive (“point neuron”) nor diffusive (“cable equation”) parts of the HH model were derived from first physical principles, but were *ad hoc* constructions based on a very simple model motivated more by its flexibility than its adherence to any first physical principles of electrodynamics. After all, a general multiparametric reaction-diffusion equation constructed with multiple time constants, thresholds, and power laws can empirically fit a multitude of physical systems, including the hypothesized neuron with multiple voltage-gated channels, even if it is not the correct physical model. That trouble was brewing should have been evident from the fact that this asynchronous, seemingly incoherent spiking activity at scales of a single neuron appeared inconsistent with observed oscillatory and wave-like patterns that are coherent across a wide range of spatial and temporal scales (Buzsaki, 2006). Attempts to reconcile these seemingly incompatible views led to the development of networks of incoherently spiking neurons (Strassberg and DeFelice, 1993; Meunier and Segev, 2002; Yamazaki et al., 2022). have However, because the original “point neuron” HH model is too complicated to describe even relatively small networks, these networks models were modified to be based on a simplified but now ubiquitous model of a leaky integrate-and-fire (LIF) neuron where a single threshold and time constant replaces all the multiple gates, currents, channels, and thresholds (Fitzhugh, 1961; Nagumo et al., 1962; Morris and Lecar, 1981; Izhikevich, 2003; Gerstner et al., 2014; Kulkarni et al., 2020; Kim and Sejnowski, 2021). The unfortunate consequence is that, rather than reconciling the two views, they now became incompatible, as LIF equations do not have a mechanism for any type of non-linear resonance to generate the sustained coherent traveling waves characteristic of neuronal “spiking” (Galinsky and Frank, 2023b). Although it is possible to obtain spatio-temporal patterns by heavily filtering the spiking data (Davis et al., 2021), the emergence of such pattern can be attributed to filtering rather than to any possible compatibility between LIF and wave models.

The source of these difficulties can be traced back to the lack of an accurate physical model of electric field dynamics that includes wave propagation and interaction in the anisotropic and inhomogeneous neural tissues. To address this deficiency, we developed such a theory that predicted the existence of previously undiscovered weakly evanescent transverse cortical brain waves (WETCOWs) generated at surfaces (or interfaces) in neural tissues as a direct consequence of their anisotropy and inhomogeneity. This theory was shown to describe a wide range of observed coherent macroscopic brain electrical activity, including extracellular spiking, hypersynchronous spiking and bursting, neuronal avalanches, and cortical wave loops (Galinsky and Frank, 2020b,a, 2021a,b), where it was shown that the networks of non-linear oscillators for those larger scale processes with the same properties—as the axonal model presented in this study—allowed for significantly more efficient synchronization than point neurons, HH-based neurons, or Kuramoto model. However, although the relationship to wave propagation in single neurons was implicit in these studies (Galinsky and Frank, 2020b,a, 2021a,b), it was not demonstrated explicitly. We do so in this current study by applying the WETCOW theory to an analytical model of a single neuron with a lipid bilayer with an anisotropic membrane conductivity. The consequence is the generation of waves of multiple frequencies and wave numbers propagating in the lipid bilayer axonal membrane that create coherent non-linear wave states consistent with the spatial-temporal characteristics of experimentally observed single neuron action potentials.

We emphasize that our theory is *not* to be interpreted as suggesting that the well-established and experimentally observed ion channel dynamics are irrelevant, but rather that they play a secondary role and that the *fundamental mechanism* of action potential generation can be attributed to the electrodynamic properties of the membrane itself. Our wave mechanism requires anisotropic current flow through the membrane. If such anisotropic current behavior can be attributed to properties other than ion channels, then our mechanism will still predict the existence of spiking. However, the specific details will depend on the specific details of the anisotropy. However, for this study, we have focused on the anisotropy created by the ion channels, as their existence and dynamics are well described, making them the easiest and the most natural way to provide this type of anisotropy. Thus, we do not claim that action potentials can emerge in membranes entirely without ion channels, but rather that the role of the ion channels may be more auxiliary than previously thought.

Of potential relevance to this view, the experimental results of time recording of extracellular waveforms reported, for example, by Gold et al. (2009) or more recently by Jung et al. (2023), have identified non-negative extracellular waveforms, such as positive and biphasic spikes, which may not directly correlate with traditional membrane potential changes. Our mechanisms can easily explain these waveforms without relying on the use of kinetic properties or even the mere presence of ion channels. We acknowledge that the HH model is not designed to model extracellular spiking, which probably means that the number of parameters in the HH model is insufficient. However, our demonstration that our more general model, which produces extracellular spiking as shown in our previous studies, also presents a simple view of the action potential, as presented in this study, is a central conclusion of this study and supports the utility of our model. It is possible to use more parameters, including all the voltages and cell geometries, to reconstruct the extracellular signal. Of course, it is always possible to keep adding even more parameters, for example, set positions and velocities of all free (and not free) electrons, ions (free and bound), charged and polar molecules, etc., across the whole brain volume and reconstruct the electric fields they produce with the highest possible accuracy and detail. However, this kind of simulation (and speculation) is outside the objectives of this study. This study aims to demonstrate that even without a more thorough simulation, the key basic properties of the action potential can be described by basic physics using a limited number of parameters.

Furthermore, having derived this directly from first principles that incorporate tissue properties, we are able to directly predict the well-known observation that signals propagate faster along myelinated axons, a result not attainable with the diffusive part of the HH model (cable equation). From a broader perspective, the demonstration that these coherent persistent traveling non-linear waves are a consequence solely of the electromagnetic properties of the neuronal tissues suggests that it may be these waves that modify states of multiple cross-membrane channels, causing them to open and close, rather the other way around, which would be a fundamental shift in the understanding of brain signaling.

We mention that although the traditional understanding of neuronal signaling has been dominated by the diffusion-reaction HH paradigm, there have been several attempts to address those deficiencies by exploring alternative approaches that incorporate the mechanical aspects of neuronal function, leading to the development of soliton-based models. These models suggest that action potentials are not purely electrical phenomena but also involve mechanical changes in the cell membrane. The soliton model, introduced by Heimburg and Jackson (2005), proposes that action potentials propagate as solitons, or solitary waves. This model suggests that the action potential is a sound pulse traveling along the axon membrane, accompanied by local changes in membrane density and thickness. Unlike the HH model, which attributes refractory periods to the dynamics of voltage-gated ion channels, the soliton theory proposes a mechanical basis for this phenomenon.

Vargas et al. (2011) explored periodic solutions and refractory periods in the soliton theory, providing insights into how mechanical aspects might influence action potential timing and frequency. Their study suggests that refractory periods emerge as a consequence of conserving the overall length and mass of the nerve, resulting in mechanical changes in membrane density and thickness that require time to recover after the passage of an action potential.

Appali et al. (2012) compared the HH model and the soliton theory, highlighting the differences in their approaches to explaining the propagation of action potentials. While the HH model relies on the sequential opening of voltage-gated ion channels, the soliton model proposes the propagation of mechanical waves. This comparison underscores the potential complementarity of these models in understanding the complex nature of neuronal signaling.

Recent study by Zhou et al. (2021) has further expanded our understanding of the electromechanical nature of neurons by investigating the role of piezoelectric sensing in auditory neurons. Their research suggests a connection between mechanical stimuli and electrical responses, potentially bridging the gap between the electrical and mechanical views of action potential propagation.

While the soliton model offers an intriguing perspective on the mechanical aspects of action potential propagation, it faces significant challenges in fully replacing the established HH model. Our model is capable of overcoming these challenges by predicting a mechanism by which low-amplitude waves can generate high-amplitude coherent propagating waves by purely electrostatic processes. In this regard, our model shows that the action potential can be described as a phase transition front (and hence the change of capacitance, which triggers ionic motion) moving along the lipid bilayer. As neurons are electrically, mechanically, and thermally coupled, our model provides a general formalism that may lead to a more comprehensive understanding of neuronal signaling that integrates both electrical and mechanical components, potentially reconciling these seemingly disparate views of action potential propagation. Due to this prediction for the action potential is based on our general model that also predicts large-scale coherences across brain regions, it bridges the gap between the local and global dynamics of observed brain activity.

## 2 Theory

The approach is similar to that developed in our general theory (Galinsky and Frank, 2020b,a, 2021a,c,b, 2023b,a,c) and is presented in this study. We begin with the general form of electromagnetic activity (Maxwell's equations), from which we derive the charge continuity equation in complex anisotropic and inhomogeneous tissues. This equation is then solved within a cylindrical geometry representation of an idealized neuron with an inhomogeneous and anisotropic membrane of finite thickness, surrounded on its inner and outer surfaces by homogeneous isotropically conducting fluids. The key here is the inclusion of a membrane conductivity tensor that provides a reasonable approximation to the electrical properties of a lipid bilayer. We then solve the simple linear problem, which demonstrates the existence of surface waves even for this reduced solution. We then extend this to the more realistic non-linear problem and demonstrate the existence of surface waves whose spatiotemporal characteristics match those of observed data of neuronal spiking, though now derived from first principles and thus directly related to neuronal geometry and microstructure.

### 2.1 The charge continuity equation

In the most general form, a description of electromagnetic activity in an axon can be formulated through Maxwell equations in a medium which is appropriate for both extracellular and intracellular regions (Scott, 1975; Bédard et al., 2004):


(1) $$\begin{array}{l} \nabla \cdot D=\rho ,\;\nabla \times H=J+\frac{\partial D}{\partial t}\;\Rightarrow \;\\ \frac{\partial \rho }{\partial t}+\nabla \cdot J=0. \end{array}$$

Using the electrostatic potential ***E*** = −∇ϕ, Ohm's law ***J*** = **σ** · ***E*** (where **σ** ≡ {σ_*ij*_} is an anisotropic conductivity tensor), a linear electrostatic property for brain tissue ***D*** = ε***E***, assuming that the scalar permittivity ε is a “good” function (i.e., it does not go to zero or infinity everywhere) and making the change of variables ∂*x* → ε*∂x*′, the charge continuity equation for the spatial-temporal evolution of the potential ϕ can be written in terms of a permittivity scaled conductivity tensor **Σ** = {σ_*ij*_/ε} as


(2) $$\begin{array}{l} \frac{\partial }{\partial t}\left(\nabla^{2}\phi \right)=-\nabla \cdot \Sigma \cdot \nabla \phi +F, \end{array}$$

where we have allowed for the influence of other sources by the inclusion of a source (or forcing) term F, that may have both linear and non-linear parts. This can be written in tensor notation as


(3) $$\begin{array}{ll} \partial_{t}\partial_{i}^{2}\phi & +\partial_{i}\left(\Sigma_{ij}\partial_{j}\phi \right)=0, \end{array}$$

where repeating indices denotes summation and the forcing term is not included (see Appendix 1 for more details).

### 2.2 Axon model

Both Equations 2, 3 are appropriate for anisotropic and inhomogeneous media in general geometry. However, for this study, it is sufficient to consider an idealized model for an axon represented by a cylindrical shell of diameter *d* created by a membrane of thickness δ/2 (that for myelinated axons includes the thickness of the myelin layers as well) that separates two homogeneous isotropically conducting fluids inside and outside of the shell with scaled conductivities $\Sigma^{i}=\sigma_{i}/\varepsilon_{i}$ and $\Sigma^{e}=\sigma_{e}/\varepsilon_{e}$. The conductivity within the thin membrane is highly anisotropic and is specified in the tensor form of the non-symmetric conductivity tensor **Σ**^*m*^, as given by Equation 11.

It should be noted that the assumption of homogeneous isotropic conductivity as well as permittivity in the external and internal fluids surrounding the cellular membranes is, of course, a standard simplification used by all diffusion cable neuron models coupled with reactive point HH sources. However, this simplification does not preclude the existence of asymmetrical and inhomogeneous distribution of charges in geometries like the membrane/fluid interface used in our model. As a matter of fact, the equilibrium solution for the electrostatic potential used as a starting point in our model necessarily requires the inhomogeneous ionic distribution in the membrane vicinity.

Experimental measurements have shown that extracellular and intracellular conductivities are similar to that of sea water (~4 S/m), or more precisely in the range from 0.28 to 2.9 S/m for extracellular σ_*e*_ and intracellular σ_*i*_ (Scott, 1975; Bédard et al., 2004), and permittivities ε_*e*_ and ε_*i*_ are around 7 × 10^−10^ F/m. Thus, both Σ^*i*^ and Σ^*e*^ are very large, on the order of 10^10^ Hz.

The conductivity of the membrane is significantly smaller. The values vary and can be assumed to be in the range from as low as 10^−13^ S/m or as high as 10^−5^ S/m (Scott, 1975), with typical values around 10^−9^ S/m (Bédard et al., 2004). With comparable or slightly smaller values for the membrane dielectric permittivity $\varepsilon_{m}\sim 10^{-11}$ F/m it gives for the membrane scaled conductivity |**Σ**| range estimate from 10^−2^ to 10^2^ Hz, hence the ratio of the conductivities of the membrane and the extracellular/intracellular media is as small as 10^−8^ to 10^−12^.

Due to this significant difference in scaled conductivities between the membrane and the surrounding fluids, for the analysis of electrodynamic processes near the membrane in the frequency range characteristic of axonal signaling it can be reasonably assumed that both extracellular and intracellular fluids act as very good (even perfect) conductors that keep the potential drop across the membrane at the resting potential value of −*V*_0_ (*V*_0_ ~65 mV). This allows using all variables normalized to the resting potential and scaled conductivity tensor of the internal fluids. Specifically, all the variables in Equations 2, 3 are normalized as *r* → *r*/*d*, $\Sigma_{ij}\to \Sigma_{ij}/\Sigma^{i}$, *t* → *t*Σ^*i*^, and ϕ → ϕ/*V*_0_. We will also introduce normalized frequencies (ω → ω/Σ^*i*^) and radial (κ → κ*d*) and axial (*k* → *kd*) wave numbers that will be used later. For the normalized membrane thickness ($\bar{\delta }\to \delta /d$), it will be assumed that $\bar{\delta }<1$. Often the difference between *d* and δ is significant so that $\bar{\delta }\ll 1$.

**Figure 1:**
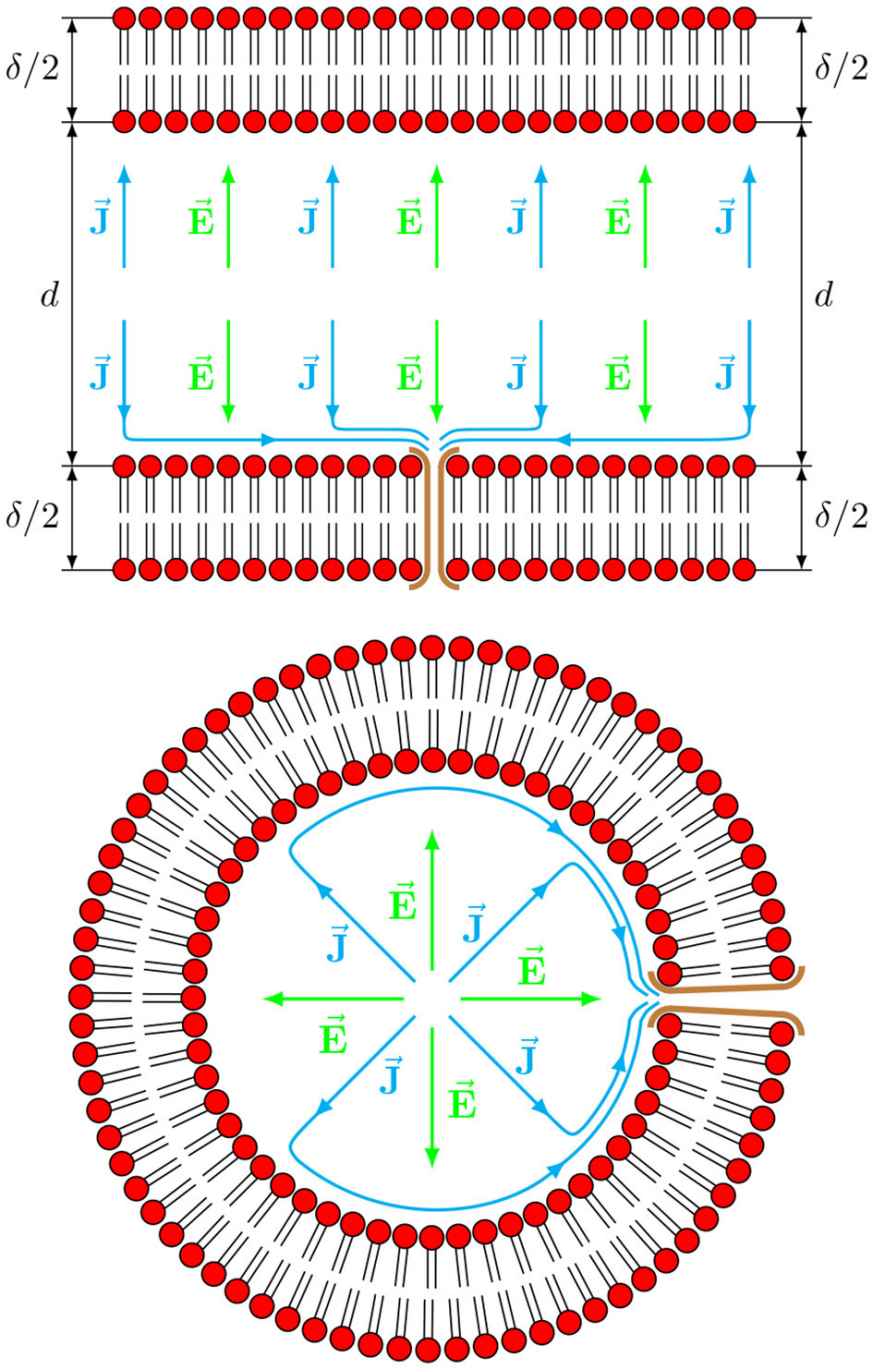
Schematic picture of an axial **(top)** and a radial **(bottom)** sections of the axon. A radial component of an electric field $\vec{E}$ inside the axon produces only a radial component of a current $\vec{J}$ with typical isotropic conductivity. However, the presence of cross-membrane channels results in the appearance of non-isotropic dependence of current as a response to a supplied electric field, giving rise to axial **(top)** and azimuthal **(bottom)** components of the current.

A simplified schematic picture of this anisotropic electric field—electric current geometry is shown in Figure 1, although it is shown not to scale as $\bar{\delta }\ll 1$ and all the anisotropic currents should be shown in the very thin boundary layer and not far outside of the $1\leq r\leq 1+\bar{\delta }$ ring. Nevertheless, the schematics can be useful to emphasize the highly anisotropic structure of the voltage-current relationship when the membrane interface is present.

### 2.3 Axon field equations

The solution to the charge continuity Equation 3 within this anisotropic and inhomogeneous axon geometry is sufficient to explain the generation of surface waves that propagate through the extracellular-intracellular membrane interface. We emphasize that this is a derivation from first physical principles, in contrast to the standard model, which is constructed from multiple empirical equations with multiple empirically fitted constants (Hodgkin and Huxley, 1952). To simplify the math in a similar fashion as our previously published study (Galinsky and Frank, 2020b) and provide a more intuitively clear result, we will assume the axon to be described by an axially symmetric cylindrical geometry (Scott, 1975), although, generally speaking, for $\bar{\delta }\ll 1$ this is not necessary.

Defining


(4) $$\begin{array}{l} u\equiv \left(\begin{array}{c} \frac{1}{r}\frac{\partial }{\partial r}r\\ \frac{\partial }{\partial z} \end{array}\right),\Sigma \equiv \left(\begin{array}{cc} \Sigma_{rr} & \Sigma_{rz}\\ \Sigma_{zr} & \Sigma_{zz} \end{array}\right),v\equiv \left(\begin{array}{c} \frac{\partial \phi }{\partial r}\\ \frac{\partial \phi }{\partial z} \end{array}\right) \end{array}$$

Equation 2 can be written for a single axon in cylindrical (*r, z*) coordinate system as (see Appendix 2 for more details)


(5) $$\begin{array}{l} \frac{\partial }{\partial t}\left(u^{t}\cdot v\right)=-u^{t}\cdot \Sigma \cdot v \end{array}$$

### 2.4 Diffusion limits of the cable theory

A standard accepted model for the propagation of the action potential spike is the so-called *cable theory*, an approach developed by Hodgkin et al. (1946) to model the passive conduction based on theoretical study on submarine telegraph cables by William Thompson (Lord Kelvin). This study was further developed and extended to dendritic spines by Rall (1962, 2011) who popularized this approach which has now become an established model in description of neuronal communication (Segev and London, 2000; Holmes, 2014; Pagkalos et al., 2023; Spruston et al., 2013) with a host of variations, such as double cables Cohen et al. (2020); Lim and Rasband (2020).

We point out that those variations, “double-cable” model in particular, strictly speaking, do not follow “HH-based formalism” in explaining the saltatory condition. Cohen et al. (2020) made an admirable attempt to salvage the diffusive-reactive (cable/Hodkin-Huxley) description using multiple interconnected RC circuits to add and mix currents/voltage drops with various phase shifts, thus effectively converting a purely diffusive cable model into a model that permits oscillatory/wave regime by creating an RC oscillator.

Due to the ubiquity and universal acceptance of the linear cable theory, we take a brief digression in this section to demonstrate that it is derivable from our more general theory, as described by Equation 5, through several simplifications. In doing so, we reveal that the standard cable theory does not support sustained propagation of the action potential in a wide range of experimentally reported physiological parameters.

#### 2.4.1 Derivation of the cable equation from Equation 5

The importance of tissue anisotropy and inhomogeneity and a complete non-linear analysis to the generation of persistent surface waves is emphasized by the fact that the cable equation, which does *not* produce such waves, can be recovered from our general model (Equation 5) by ignoring important components that contribute to these properties, namely, the non-diagonal and non-linear terms in the conductivity tensors.

Ignoring all non-diagonal and non-linear terms in the conductivity tensors and assuming that only $\Sigma_{⊥}^{0}$ and $\Sigma_{∥}^{0}$ terms are non-zero, so that **Σ** in Equation 4,


(6) $$\begin{array}{l} \Sigma \equiv \left(\begin{array}{cc} \Sigma_{⊥}^{0} & 0\\ 0 & \Sigma_{∥}^{0} \end{array}\right), \end{array}$$

the equation for the electric field potential from Equation becomes 5,


(7) $$\begin{array}{l} \frac{\partial }{\partial t}\left(\frac{1}{r}\frac{\partial }{\partial r}r\frac{\partial \phi }{\partial r}+\frac{\partial^{2}\phi }{\partial z^{2}}\right)=-\Sigma_{⊥}^{0}\frac{1}{r}\frac{\partial }{\partial r}r\frac{\partial \phi }{\partial r}-\Sigma_{∥}^{0}\frac{\partial^{2}\phi }{\partial z^{2}}. \end{array}$$

As cable equation is not supposed to follow the exact radial dependence of the ϕ, we can use Equation 7 and obtain its approximate form in the limit of a very thin lipid bilayer, that is, $\bar{\delta }\ll 1$ and assuming that the largest radial variations of the potential ϕ are located around the membrane. This enables an approximate solution where the time dependence of the field is wholly contained in the axial dimension. At the same time, the radial component is constructed to meet some minimal boundary conditions based on simple geometric constraints. Therefore, we can search for the approximate solution separable in the radial and axial dimensions of the form $\phi =\phi_{r}^{\prime }\left(r\right)\phi_{a}\left(z,t\right)$, where $\phi_{r}^{\prime }\left(r\right)$ is equal to −1 for 0 ≤ *r* ≤ 1, transitions from -1 to 0 for $1\leq r\leq 1+\bar{\delta }$, that is, $\phi_{r}^{\prime }\left(r\right)=-1+\ln r/\ln\left(1+\bar{\delta }\right)$, and equals 0 for $r>1+\bar{\delta }$. Multiplying Equation 7 by $\phi_{r}^{\prime }\left(r\right)r$ and integrating it from 0 to infinity, we obtain the cable equation in the usual form (Rall, 2011; Holmes, 2014) as


(8) $$\begin{array}{l} \frac{1}{\bar{\delta }}\frac{\partial \phi_{a}}{\partial t}+\frac{\Sigma_{⊥}^{0}}{\bar{\delta }}\phi_{a}=\frac{\Sigma_{∥}^{0}}{2}\frac{\partial^{2}\phi_{a}}{\partial z^{2}}, \end{array}$$

where we used


(9) $$\begin{array}{l} \overset{\infty }{\underset{0}{\int }}\frac{1}{r}\frac{\partial }{\partial r}r\frac{\partial \phi }{\partial r}\phi_{r}^{\prime }\left(r\right)rdr=-\phi_{a}\overset{1+\bar{\delta }}{\underset{1}{\int }}|\frac{\partial \phi_{r}^{\prime }}{\partial r}{|}^{2}rdr\approx -\frac{\phi_{a}}{\bar{\delta }}, \end{array}$$

(10) $$\begin{array}{l} \overset{\infty }{\underset{0}{\int }}\phi \phi_{r}^{\prime }\left(r\right)rdr=\phi_{a}\overset{1+\bar{\delta }}{\underset{0}{\int }}|\phi_{r}^{\prime }\left(r\right){|}^{2}rdr\approx \frac{\phi_{a}}{2}. \end{array}$$

and have ignored the second term under time derivative in Equation 7 because it is negligible when the axial scales of variation of the potential ϕ_*a*_ is larger than $\sqrt{\bar{\delta }/2}$. We thus recover the cable Equation 8 from 7, where $\Sigma_{⊥}^{0}$ corresponds to the normalized membrane scaled conductivity and $\Sigma_{∥}^{0}$ equals to the normalized scaled conductivity of the axon internal fluid (i.e., $\Sigma_{∥}^{0}\sim$ 1 in dimensionless units). The terms in Equation 7 from Equation 8 directly correspond to dissipative (no positive feedback) terms of Λ_⊥_ (Equations 20–22) in the limit $k^{2}\ll \kappa^{2}\sim 1/{\bar{\delta }}^{2}$.

#### 2.4.2 Length and time scale of the cable equation

The cable equation describes that the height of the action potential peak decays with time *t* as $\sqrt{t_{0}/t}$, where the shortest time $t_{0}\sim 1/\Sigma_{∥}^{0}$ corresponds to the narrowest ($\sqrt{\bar{\delta }/2}$) and the tallest (ϕ_*a*_(*t*_0_, *z*_0_) = ϕ_*m*_) shape of the action potential peak that the cable equation is capable of describing (and when the cable equation approximation is valid). The decay is actually even faster as it includes an exponential term $\exp\left(-\Sigma_{⊥}^{0}t\right)$, but the approximate time dependence will be valid when $t<1/\Sigma_{⊥}^{0}$ and, as the ratio of the cross-membrane to the intracellular conductivities is very small (10^−8^ to 10^−12^) (Bédard et al., 2004), we can safely use this approximation in all our estimates below. The dimensionless diffusion coefficient is then equal to $\Sigma_{∥}^{0}\bar{\delta }/2$, which allows us to find the time dependence of the axial diffusion length (the half-width of the pulse) as $\Delta z\sim \sqrt{t\Sigma_{∥}^{0}\bar{\delta }/2}$.

The ratio of the differences of the action potential firing threshold to the total peak above the resting potential in the “point neuron” HH-model is equal to approximately Δϕ_*a*_/ϕ_*m*_ ~ 0.15 (resting potential is –70 mV, threshold –55 mV, peak 30 mV). Therefore, the maximum time until the diffusively spreading pulse reaches the threshold is approximately $\sim t_{0}\phi_{m}^{2}/{\Delta \phi_{a}}^{2}\ll 1/\Sigma_{⊥}^{0}$, giving the maximum diffusion length $\Delta z\sim \sqrt{\bar{\delta }/2}\phi_{m}/\Delta \phi_{a}$.

#### 2.4.3 Myelinated axons

For a myelinated 20 μm diameter axon with the thickest myelin layer ($\bar{\delta }\sim 0.4$) this gives the maximum diffusion length of approximately 60 μm, which is significantly shorter than the internodal length of ~ 2mm between the Nodes of Ranvier of the typical 20 μm axon. Decreasing the myelin thickness will further reduce the maximum propagation length. Naïve attempts to adjust the threshold parameters of the “point neuron” HH-model to accommodate a longer maximum diffusion length will quickly reveal significant model inconsistencies. For example, using the typical average ranges of internodal distances for different axon diameters (350 μm for 12 μm axon diameter, 205 μm for 3.4 μm axon diameter (Waxman and Melker, 1971), to 139 μm for 0.82 μm axon diameter (Arancibia-Cárcamo et al., 2017)) it can be easily seen that it will require to decrease the firing threshold in 10–60 times ($350\times 0.15/12/\sqrt{\bar{\delta }/2}\sim 10$, $205\times 0.15/3.4/\sqrt{\bar{\delta }/2}\sim 20$, $139\times 0.15/0.82/\sqrt{\bar{\delta }/2}\sim 60$), hence will require exceedingly (and unrealistically) low-threshold voltages in the range of -68.5 to -69.75 mV instead of -55 mV for the resting potential of –70 mV. The conclusion is therefore that the amount of diffusion provided by standard cable theory for action potential spike generation by thresholded reactive “point neuron” HH mechanism with experimentally confirmed parameter values is generally incompatible with the process of saltatory conduction.

#### 2.4.4 Unmyelinated axons

For unmyelinated axon the original “point neuron” HH model assumes that there are 60 Na^+^ channels and 18 K^+^ channels for every μm^2^ of membrane (Sengupta et al., 2013). A more detailed analyses of variations of Na^+^ channels density show that in unmyelinated hippocampal axons, the density increases tenfold from the soma with 2.6 channels/μm^2^, through the proximal axon (25 channels/μm^2^), to the distal axon (46.1 channels/μm^2^) (Hu and Jonas, 2014; Freeman et al., 2016). Therefore, it can be safely assumed that the average linear distance between ion channels for an unmyelinated axon change from approximately 0.1 μm to 0.7 μm ($\sim 1/\sqrt{2.6}$). For a 500 μm diameter giant squid axon with 10 nm membrane, like the one used by Hodgkin and Huxley in their seminal work, the maximum diffusion length is approximately $\sqrt{10^{-2}/2/500}\times 500/0.15\sim 10.5\mu$m, which is significantly above the average linear inter-channel distance of 0.1 μm, thus gives enough flexibility to successfully do a mind entertaining exercise of fitting diffusive and reactive processes together. However, for thin unmyelinated pyramidal tract dendrites with around 0.2 μm diameter and 5-nm membrane thickness ($\bar{\delta }\sim 0.025$), the maximum propagation distance is approximately 0.15 μm, that is, several times less than 5 Na^+^ channels/μm^2^ and 5 K^+^ channels/μm^2^ density of pyramidal neuron (Arhem et al., 2006) would provide.

The conclusion is that the action potential propagation model described by cable theory is incompatible with experimentally measured physiological parameters of both myelinated and unmyelinated axons. On the contrary, our linear wave model, developed from first principles using measured physiological tissue parameters, is capable of describing wave propagation across all these parameter ranges, as will be demonstrated in the sections that follow.

### 2.5 The conductivity tensor of the lipid bilayer

As shown in our previous study (Galinsky and Frank, 2020b), the existence of electric field surface waves is predicated on the inhomogeneity and anisotropy of the neural tissues. Remarkably, though, this does not require an exceedingly accurate characterization of tissue microstructure. Instead, local average tissue parameterizations are sufficient to make accurate predictions of complex regional and long-range non-linear wave propagation properties. This is an important point, as a substantial body of literature suggests the need for highly precise, complex tissue models to accurately predict observed coherent macroscopic electromagnetic brain activity. As demonstrated in our previous publications Galinsky and Frank (2020b,a, 2021a,c,b, 2023b,a,c), this is not the case.

The same holds true for the single axon case considered here, where a reasonable model for the membrane conductivity tensor **Σ**^*m*^ can be constructed using a set of fairly general assumptions, resulting in the generation of surface waves in the lipid membrane. First, it is assumed that both the along-axon (i.e., axial *z*) and across-axon (i.e., radial *r*) electric fields will generate currents not only along the electric filed direction (i.e., along *z* and *r*, respectively) but will also generate currents that are perpendicular to the field (i.e., along *r* and *z*, respectively) due to anisotropy and inhomogeneity of axonal geometry, as shown in Figure 1. That is, for radial (along *r*) fields Σ_*zr*_ ≠ 0 and for axial (along *z*) fields Σ_*rz*_ ≠ 0. Based only on these symmetry considerations, the membrane conductivity tensor **Σ**^*m*^ is assumed to have the following non-diagonal, non-symmetric form


(11) $$\begin{array}{l} \Sigma^{m}=\left(\begin{array}{cc} \Sigma_{rr}\left(\text{Ψ}\right) & \Sigma_{rz}\left(\text{Ψ}\right)\\ \Sigma_{zr}\left(\text{Ψ}\right) & \Sigma_{zz}\left(\text{Ψ}\right) \end{array}\right), \end{array}$$

where we indicate the fact that Ohm's law inside the membrane is non-linear by adding a dependence of the conductivity tensor components on some functional form Ψ[ϕ] of scalar potential ϕ (that may be either scalar Ψ = ϕ, or vector Ψ = −∇ϕ, or even more complex). The currents generated parallel to the field are not expected to be equal (i.e., Σ_*rr*_ ≠ Σ_*zz*_) nor would be equal the currents generated perpendicular to the field (Σ_*rz*_ ≠ Σ_*zr*_).

### 2.6 Solutions to the field equations

Due to the vast difference in scaled conductance between the inside and outside the bilayer membrane, the assumption of a perfectly conducting boundary condition on both sides of the membrane bilayer is accurate, and explicit solutions for the extracellular and intracellular spaces are not required. It is only necessary to solve Equation 5 for inside the ring $1\leq r\leq 1+\bar{\delta }$. We will seek the solution in the form


(12) $$\begin{array}{l} \phi \left(r,z,t\right)=\phi^{0}\left(r\right)+\phi^{\prime }\left(r,z,t\right), \end{array}$$

(13) $$\begin{array}{l} \phi^{0}\left(r\right)=\frac{\ln\;r}{\ln\;\left(1+\bar{\delta }\right)}\approx \frac{1}{\bar{\delta }}\ln\;r, \end{array}$$

where ϕ^0^(*r*) is a stationary, time independent (or equilibrium) solution of the Equation 5 inside the ring $1\leq r\leq 1+\bar{\delta }$, such that ϕ^0^(*r*) ≤ 1 anywhere inside the ring whereas outside the ring ϕ^0^(*r*) = 0 for *r* ≤ 1 and ϕ^0^(*r*) = 1 for $r\geq 1+\bar{\delta }$ (see Appendix 3.1 for more details).

The solution to the field equations can be approached at two levels of accuracy, a simplified but intuitive linear version, and a more accurate but complex non-linear version, by formally expanding the non-linear dependence of the conductivity tensor in dimensionless form into a Taylor series as


(14) $$\begin{array}{l} \Sigma (\Psi )=\Sigma^{0}+\Sigma^{\prime }(\Psi -{\bar{\Psi }}_{0})+\ldots , \end{array}$$

(15) $$\begin{array}{l} \Sigma^{0}\equiv \Sigma (\Psi ){|}_{\Psi ={\bar{\Psi }}_{0}}\text{ and }\Sigma^{\prime }\equiv {\frac{\partial \Sigma (\Psi )}{\partial \Psi }|}_{\Psi ={\bar{\Psi }}_{0}} \end{array}$$

where ${\bar{\text{Ψ}}}_{0}$ should be taken as an average across the membrane for any particular functional form of equilibrium potential dependence, that is ${\bar{\text{Ψ}}}_{0}=\bar{\text{Ψ}\left[\phi^{0}\left(r\right)\right]}$. Equation 14 has been constructed with an adjustable normalization *V* to facilitate the inclusion of external conditions such as those prevalent in a wide range of experiments. For example, it can be set to the equilibrium value of a voltage drop across the membrane for voltage-clamping experiments.

Without a loss of generality we can assume that the zeroth order terms are axisymmetric, with average dimensionless cross-membrane conductivity $0<\Sigma_{rr}^{0}\equiv \Sigma^{0}\ll 1$, average conductivity along the membrane (that possibly is significantly smaller) $\Sigma_{zz}^{0}=\epsilon \Sigma_{rr}^{0}$ (ϵ < 1), and zero off-diagonal terms $\Sigma_{rz}^{0}=\Sigma_{zr}^{0}=0$, that is,


(16) $$\begin{array}{l} \Sigma^{0}=\Sigma^{0}\left(\begin{array}{cc} 1 & 0\\ 0 & \epsilon \end{array}\right). \end{array}$$

With this positive definite matrix form used for **Σ**^0^, the only solution that can be obtained from Equation 5 corresponds to the loss of electrostatic field energy in the membrane. To compensate for this loss and maintain the potential difference across the membrane at a fixed “resting potential” level, additional mechanisms are required. In axons, this occurs through the addition of energy via adenosine triphosphate (ATP) mediated diffusion. For this study, we are not interested in the details of this process, and we will assume that it provides the required amount of energy to maintain a constant level of the cross-membrane voltage drop. This means that our model incorporates both passive and active membrane properties, but active properties are included only implicitly. We consider that active transport is available and conduct the necessary study to maintain the average potential drop across the membrane at an appropriate level.

Due to the different concentrations of the different ions in extracellular and intracellular fluids (in particular, sodium and potassium ions), it has been known for a long time that non-linear membrane properties show a positive feedback effect for the radial current-voltage relationship (Scott, 1975). In terms of the non-linear passive response produced by the conductivity tensor, it means that some of the **Σ**^′^ components are negative. At the same time the structure of **Σ**^′^ should guarantee that there is neither total (volume integrated) additional electrostatic energy loss nor total electrostatic energy generation produced due to this non-linear self coupling, therefore both eigenvalues of **Σ**^′^ should be zeros (the eigenvalues of the conductivity matrix are real, see Appendix 4 for more details). As membrane conductivity is normalized by Σ^*i*^, we would require that |Σ_{… }_| ≤ 1, and will assume that both $\left|\Sigma_{\left\{\ldots \;\right\}}^{0}\right|$ and $\left|\Sigma_{\left\{\ldots \;\right\}}^{\prime }\right|$ are less than 1. This limits the structure of **Σ**^′^ to the following form


(17) $$\begin{array}{l} \Sigma^{\prime }=\Sigma^{\prime }\left(\begin{array}{cc} s_{⊥}xy & -s_{∥}x^{2}\\ s_{∥}y^{2} & -s_{⊥}xy \end{array}\right), \end{array}$$

where $\Sigma^{\prime }=\max|\Sigma_{ij}^{\prime }|$, max(*x, y*) = 1, min(*x, y*) ≥ 0, and both *s*_⊥_ and *s*_∥_ can either be –1 or 1. As we will see below, the choice of *s*_∥_ between –1 and 1 is not particularly important, as it simply selects different directions of wave propagation. Still, the different choice for a sign of *s*_⊥_ selects different scales where wave excitation and/or damping occur, which has been experimentally noted as different behaviors of spiking for Type I and Type II neurons.

Based on experimental results that we cited above (Scott, 1975; Bédard et al., 2004), the normalized linear membrane conductivity is expected to be significantly less than 1 ($|\Sigma_{\left\{\ldots \;\right\}}^{0}|\sim 10^{-8}-10^{-12}\ll 1$). Therefore, the assumption for the first order normalized membrane conductivity that $|\Sigma_{\left\{\ldots \;\right\}}^{\prime }|<1$ does not require it to be smaller than the linear normalized membrane conductivity, on the contrary it may be expected that $1>|\Sigma_{\left\{\ldots \;\right\}}^{\prime }|\gg |\Sigma_{\left\{\ldots \;\right\}}^{0}|$.

The solution for the second term ϕ′(*r, z, t*) in Equation 12 can be expanded using radial and axial eigenmodes of the linearized system, with perfectly conducting boundary conditions at *r* = 1 and $r=1+\bar{\delta }$ that require that *E*_*z*_ = 0 or $\phi_{r}\left(1\right)=\phi_{r}\left(1+\bar{\delta }\right)=0$. Those functions are


(18) $$\begin{array}{l} \phi_{r}\left(r\right)\sim R_{0}\left(\kappa r+\eta \right),\;\phi_{w}\left(z,t\right)\sim e^{-i\left(\omega_{k}t+kz\right)}, \end{array}$$

where *R*_0_ denotes Bessel functions either of the first (*J*_0_) or the second (*Y*_0_) kind, and κ and η can be determined from the boundary conditions, $R_{0}\left(\kappa +\eta \right)=R_{0}\left(\kappa \left(1+\bar{\delta }\right)+\eta \right)=0$. Note that the parameters κ in ϕ_*r*_(*r*) plays a similar role as the axial wave number *k* in ϕ_*w*_(*z, t*) as larger values produce shorter wavelength spatial oscillations, but in the radial direction (although we are not interested in different radial modes and assume an existence of the longest mode with $\kappa \bar{\delta }\sim 1$). The derivative of the radial eigenmode can then be written as


(19) $$\begin{array}{l} \frac{d\phi_{r}}{dr}\sim -\kappa R_{1}\left(\kappa r+\eta \right), \end{array}$$

where again *R*_1_ denotes Bessel functions either of the first (*J*_1_) or the second (*Y*_1_) kind, and $R_{1}\left(\kappa +\eta \right)\approx \pm R_{1}\left(\kappa \left(1+\bar{\delta }\right)+\eta \right)$ (see Appendix 3.2 for more details).

Proceeding in a spirit similar to our earlier analysis (Galinsky and Frank, 2020b), we first solve the simpler linear wave analysis problem by considering only the linear terms in Equation 14 which are independent of *z* and *t*, then expand the scope of the analysis to include the non-linear terms that depend on *z* and *t*.

### 2.7 Linear wave analysis and surface wave generation

The linear in ϕ′(*r, z, t*) terms in Equation 5 that are independent of *z* and *t* include from Equation 14 $\Sigma_{\left\{\ldots \;\right\}}^{0}$, that are constant inside the membrane layer, and $\Sigma_{\left\{\ldots \;\right\}}^{\prime }\left(\text{Ψ}\left[\phi^{0}\right]-{\bar{\text{Ψ}}}_{0}\right)$, that only depend on radius *r*. Substituting the eigenmode solutions (Equation 18) into Equation 5, multiplying by ϕ_*r*_(*r*)*r*, and integrating the radial part across the membrane bilayer, we obtain the complex dispersion relation (see Appendix 5 for more details)


(20) $$\begin{array}{ll} i\Omega_{k} & \equiv \gamma_{k}+i\omega_{k}=\Lambda_{⊥}+i\Lambda_{∥}k \end{array}$$

and the real Λ_⊥_ and the imaginary *i*Λ_∥_*k* parts of the dispersion correspond to the diagonal and the off diagonal conductivity tensor components,


(21) $$\begin{array}{ll} \gamma_{k} & \equiv \Lambda_{⊥}=\left(\gamma_{d}-\gamma_{e}\right), \end{array}$$

(22) $$\begin{array}{l} \gamma_{d}=\frac{\Sigma^{0}}{\varkappa^{2}}\left(\kappa^{2}+\epsilon k^{2}\right), \end{array}$$

(23) $$\begin{array}{l} \gamma_{e}=\hat{V}\Sigma^{\prime }\frac{s_{⊥}xy}{\varkappa^{2}}\frac{\left(\kappa^{2}C_{⊥}^{r}-k^{2}C_{⊥}^{z}\right)}{C} \end{array}$$

(24) $$\begin{array}{l} \;\approx \hat{V}\Sigma^{\prime }\frac{s_{⊥}xy}{2\varkappa^{2}}\left(\kappa^{2}-k^{2}\right), \end{array}$$

(25) $$\begin{array}{l} \Lambda_{∥}=\hat{V}\Sigma^{\prime }\frac{C_{∥}}{2C}\frac{s_{∥}\left(x^{2}+y^{2}\right)}{\varkappa^{2}}\approx \hat{V}\Sigma^{\prime }\frac{s_{∥}\left(x^{2}+y^{2}\right)}{2\bar{\delta }\varkappa^{2}}, \end{array}$$

where we introduced an adjustable normalization *V* to facilitate the inclusion of external conditions such as those prevalent in a wide range of experiments, for example, it can be set to the equilibrium value of a voltage drop across the membrane for voltage-clamping experiments. In Equations 23, 25 $\hat{V}\equiv V/V_{0}$ is the fractional voltage (i.e., the fraction of the resting potential occupied by the external voltage) and


(26) $$\begin{array}{ll} \varkappa^{2} & \equiv \kappa^{2}+k^{2} \end{array}$$

The normalization parameters *C*_⊥_, *C*_∥_, and *C* are provided in Appendix 5 in Equations 105–108 and also in Appendix 6. The parameter ϰ^2^ can be viewed as the length (squared) of a vector ***ϰ*** = ***κ*** + ***k*** in an abstract vector space that controls the spatial scale of oscillations in the radial and longitudinal (axial) coordinates of the axon. The component Λ_⊥_ describes the damping (γ_*d*_) or excitation (γ_*e*_) of the waves while Λ_∥_ is related to the wave oscillations ω_*k*_. Equations 21, 23 can be approximated as


(27) $$\begin{array}{ll} \Lambda_{⊥} & \approx \Sigma^{0}\left[\left({\hat{\kappa }}^{2}+\epsilon {\hat{k}}^{2}\right)+{\hat{\sigma }}_{⊥}\left({\hat{k}}^{2}-{\hat{\kappa }}^{2}\right)\right] \end{array}$$

(28) $$\begin{array}{ll} \Lambda_{∥} & \approx \Sigma^{0}\frac{{\hat{\sigma }}_{∥}}{\bar{\delta }\varkappa^{2}}, \end{array}$$

where $\hat{\kappa }\equiv \kappa /\varkappa$ and $\hat{k}\equiv k/\varkappa$ are the fractional wave numbers and


(29) $$\begin{array}{ll} {\hat{\sigma }}_{⊥} & \equiv \frac{1}{2}\hat{V}\hat{\Sigma }s_{⊥}xy \end{array}$$

(30) $$\begin{array}{ll} {\hat{\sigma }}_{∥} & \equiv \frac{1}{2}\hat{V}\hat{\Sigma }s_{∥}\left(x^{2}+y^{2}\right) \end{array}$$

where $\hat{\Sigma }\equiv \Sigma^{\prime }/\Sigma_{0}$ is the fractional conductivity (i.e., the ratio of the conductivity perturbation magnitude to the mean membrane conductivity). The parameters ${\hat{\sigma }}_{⊥}$ and ${\hat{\sigma }}_{∥}$ are the weightings for the (fractional) radial and longitudinal wave vector contributions to the radial and parallel components, respectively, of the dispersion relation. Each is scaled by both the fractional voltage and the fractional conductivity. The radial and longitudinal are scaled, respectively, by *s*_⊥_ = ±1 and *s*_∥_ = ±1 that have been introduced to demonstrate the profoundly different wave characteristics possible within the available parameter ranges defined in Equation 20.

#### 2.7.1 The existence of waves

This solution to the simplified linear problem is sufficient to demonstrate a key result: the existence of propagating surface waves along the axon. To see this, note that for large *k* (*k* ≫ κ) that ϰ ≈ *k* so from Equation 28, $\Lambda_{∥}\sim 1/k^{2}$ so that the oscillatory component of the dispersion relation (Equation 20) is approximately ω_*k*_ = Λ_∥_*k* ~ 1/*k* and thus exhibits the same inverse proportionality of frequency and wave number shown in our previous study Galinsky and Frank (2020b,a) (using Cartesian geometry) to generate surface (or interface) electric field waves. The relative magnitude of the conductivity tensor components defined in Equation 14 are such that $1>|\Sigma_{\left\{\ldots \;\right\}}^{\prime }|\gg |\Sigma_{\left\{\ldots \;\right\}}^{0}|$ so that $\hat{\Sigma }\gg 1$ and thus the fractional conductivities defined in Equations 29, 30 provide sufficiently large parameter ranges within the membrane to support wave excitation.

#### 2.7.2 Wave characteristics

The parameters *s*_∥_ = ±1 and *s*_⊥_ = ±1 were introduced to delineate the profoundly different parameter regions of the dispersion relation (Equation 20). This can now be shown directly using Equations 28, 27.

First, consider the parallel component *s*_∥_. Note that the phase velocity of the waves is defined as ω_*k*_/*k* = Λ_∥_, and thus it is determined by Equation 28. Changing the sign of *s*_∥_ changes the sign of σ_∥_ in Equation 30 and thus changes the sign of the phase velocity (Equation 28). That is, it changes the direction of the wave propagation.

Now consider the perpendicular component *s*_⊥_. Its influence on the solution provides an important new understanding of the role of the Nodes of Ranvier. Changing the sign of *s*_⊥_ changes the sign of the wave excitation rate γ_*e*_ (Equation 23), resulting in two distinct wave excitation patterns. If *s*_⊥_ is positive, γ_*e*_ > 0 when *k* < κ, that is, waves with longer wavelength will be excited, which corresponds to Type I myelinated axons, where longer wavelengths are preset by the internodal distances between the Nodes of Ranvier and the maximum wavelength will be determined by the strongest excitation at the internodal length. For *s*_⊥_ = −1 the wave excitation rate γ_*e*_ will be positive for *k* > κ and will be increasing with the increase of the wave number *k*, hence shorter scale waves (often at the subthreshold level) and higher frequencies will be seen, that is, more representative of unmyelinated Type II (and possibly some unmyelinated Type I as well) behavior.

### 2.8 Wave speeds and myelination

The dispersion relation enables the calculation of the wave phase velocity, which is the rate at which a wave of a single frequency propagates through the medium. The dimensional wave phase speed V for the component along the axon from Equation 20 is


(31) $$\begin{array}{l} V\equiv \frac{\omega_{k}}{k}\Sigma^{i}d=\Lambda_{∥}\Sigma^{i}d \end{array}$$

where the factor Σ^*i*^*d* appeared as the parameters have been converted to dimensional form. The simple estimates of wave phase velocity, particularly the dependence of the velocity on axon diameter *d*, exhibit consistent behavior across both myelinated and unmyelinated conditions.

#### 2.8.1 Myelinated axons

For myelinated axons, the ratio of the axon diameter to the total (axon and myelin) diameter is relatively constant (around 0.6–0.8) (Gillespie and Stein, 1983; Arancibia-Cárcamo et al., 2017) so that in our dimensionless units ${\bar{\delta }}_{m}\sim 0.2-0.4$. This determines the radial oscillation spatial wave number $\kappa_{m}\sim \pi /{\bar{\delta }}_{m}\sim 5-15$. As myelination reduces the cross-membrane conductivity ($\Sigma_{rr}^{0}$), it effectively decreases the wave damping γ_*d*_ for all scales smaller than the inter-node distance. Therefore, we may assume that the inter-node distance between the Nodes of Ranvier *L*_*m*_ determines the wavelength of the propagating modes. The inter-node distance between Nodes of Ranvier *L*_*m*_ can be as high as 1.5 mm, but typically ranges from 350 μm for 12 μm axon diameter, to 205 μm for 3.4 μm axon diameter (Waxman and Melker, 1971), to 139 μm for 0.82 μm axon diameter (Arancibia-Cárcamo et al., 2017) so that parallel spatial wave number *k*_*m*_ ~ 2π/*L*_*m*_ ~ 0.005 − 0.05 and κ ≫ *k*_*m*_ so that ϰ ≈ κ. Hence, for myelinated axons the wave phase speed is directly proportional to axonal diameter (assuming that $\Sigma_{∥}^{\prime }=\left(x^{2}+y^{2}\right)\hat{V}\Sigma^{\prime }/2\sim 0.05$, that is, less than a maximum value of 1 due to multiple layers of myelin, *d* is in the units of μm, and a conversion factor from μm to *m* is included into the numerical constant)


(32) $$\begin{array}{l} V=\Lambda_{∥}\Sigma^{i}d\sim 5\times 10^{3}\frac{\Sigma_{∥}^{\prime }}{{\bar{\delta }}_{m}\kappa_{m}^{2}}d\sim \left(5-10\right)d \end{array}$$

in units of *m*/*s*, giving values of 100–200 m/s for 20 μm diameter axons which is consistent with published values (Siegel and Sapru, 2005).

These results also explain some recently detected anomalous phenomena of nerve conduction, such as the observation that in myelinated nerves, the conduction velocity increases with stretch, which contradicts existing theories (see, e.g., Schmidt and Knösche, 2019) since the diameter decreases upon stretching (Sharmin et al., 2023). However, this agrees well with our results as stretching increases the intra-nodal distance, hence increases both the wavelength and the wave phase velocity (Equation 28).

#### 2.8.2 Unmyelinated axons

For unmyelinated axons the membrane diameter is constant δ_*u*_ ~ 10 nm = 10^−2^μm and the wavelength *L*_*u*_ of the propagating modes is going to be significantly smaller (depending on the small-scale membrane geometry), but it is reasonable to assume *L*_*u*_ ~ *d*/10. That gives for the dimensionless wavenumber $k_{u}\sim 2\pi d/L_{u}\sim 20\pi \sim 10^{2}$, and κ_*u*_ is again determined by the same relation $\kappa_{u}\sim \pi /{\bar{\delta }}_{u}$, where ${\bar{\delta }}_{u}$ now is not fixed, ${\bar{\delta }}_{u}=\delta_{u}/d$. Then the expression to the wave speed as a function of *d* (assuming maximum value for $\Sigma_{∥}^{\prime }\sim 1$, and both *d* and δ_*u*_ in the units of μm)


(33) $$\begin{array}{l} V=\Lambda_{∥}\Sigma^{i}d\sim 5\times 10^{3}\frac{\Sigma_{∥}^{\prime }}{\delta_{u}k_{u}^{2}}\frac{d^{2}}{1+d^{2}\pi^{2}/\left(\delta_{u}^{2}k_{u}^{2}\right)}, \end{array}$$

giving roughly a range 0.5—5 m/s for axon diameters, ranging 0.1—10 μm. Thus, the wave speeds of myelinated axons are predicted to be around two orders of magnitude larger than those of unmyelinated axons. The importance of this analysis lies not only in the consistency of these predictions with measured values, but also in the fact that they were derived from first principles and are therefore based on rather simple (at least to first order) measurable axon characteristics. This offers the potential for a better understanding of brain communication deficits associated with ubiquitous demyelinating diseases such as multiple sclerosis.

### 2.9 Non-linear wave analysis

The linear wave analysis above is sufficient to demonstrate the existence of sustained propagating waves along the axons. However, as demonstrated in our previous study (Galinsky and Frank, 2020b,a), a full non-linear analysis is necessary to accurately describe the details of the spatiotemporal characteristics of the propagating waves.

Proceeding as in Galinsky and Frank (2020b,a), the solution ϕ_*w*_(*z, t*) is expanded using a Fourier integral


(34) $$\begin{array}{l} \phi_{w}\left(z,t\right)=\overset{\infty }{\underset{-\infty }{\int }}a_{k}\left(t\right)e^{i\left(kz+\omega_{k}t\right)}dk+c.c., \end{array}$$

assuming that


(35) $$\begin{array}{l} \left|\frac{1}{a_{k}\left(t\right)}\frac{da_{k}\left(t\right)}{dt}\right|<\omega_{k}. \end{array}$$

and where c.c. refers to the complex conjugate. This results in a set of coupled equations for time-dependent complex amplitudes *a*_*k*_(*t*)≡*a*(*k, t*)


(36) $$\begin{array}{l} \frac{da_{k}}{dt}=\left(\gamma_{e}-\gamma_{d}\right)a_{k}+N_{k}, \end{array}$$

that have the same general form as Equation 14 in Galinsky and Frank (2020b), where


(37) $$\begin{array}{l} N\left(\phi \right)=D_{⊥}\phi_{w}^{2}+D\frac{d\phi_{w}^{2}}{dz}+D_{∥}\frac{d^{2}\phi_{w}^{2}}{dz^{2}}, \end{array}$$

(38) $$\begin{array}{l} N_{k}=\frac{1}{2\pi }\overset{\infty }{\underset{-\infty }{\int }}N\left(\phi \right)e^{-i\left(kz+\omega_{k}t\right)}dz, \end{array}$$

where the normalization coefficients *D*_⊥_, *D*_∥_, and *D* are given in Appendix 7 (see Equation 117). The detailed evaluation of non-linear input from multiple wave modes, assuming a general quadratic form of non-linearity, was shown in detail for both non-resonant and resonant terms in (Galinsky and Frank, 2020b,a). It was shown there for the first time that it is the inverse proportionality between frequencies and wave modes that allows calculation of the non-linear input in a relatively simple analytical form, resulting in a simple non-linear equation for wave amplitude *a*_*k*_(*t*). Following (Galinsky and Frank, 2021c, 2023a,c) this equation can be written in the general form


(39) $$\begin{array}{l} \frac{da_{k}}{dt}=\gamma_{k}a_{k}+\beta_{k}^{\prime }a_{k}a_{k}^{*}+\beta_{k}a_{k}^{2}-\alpha_{k}a_{k}{\left(a_{k}a_{k}^{*}\right)}^{1/2}, \end{array}$$

where complex γ_*k*_ includes γ_*e*_ − γ_*d*_ as a real part and ω_*k*_ as an imaginary part, and the parameters α, β, and β′ can be evaluated following (Galinsky and Frank, 2020b,a) using coefficients provided in Equations 36, 37.

**Figure 2:**
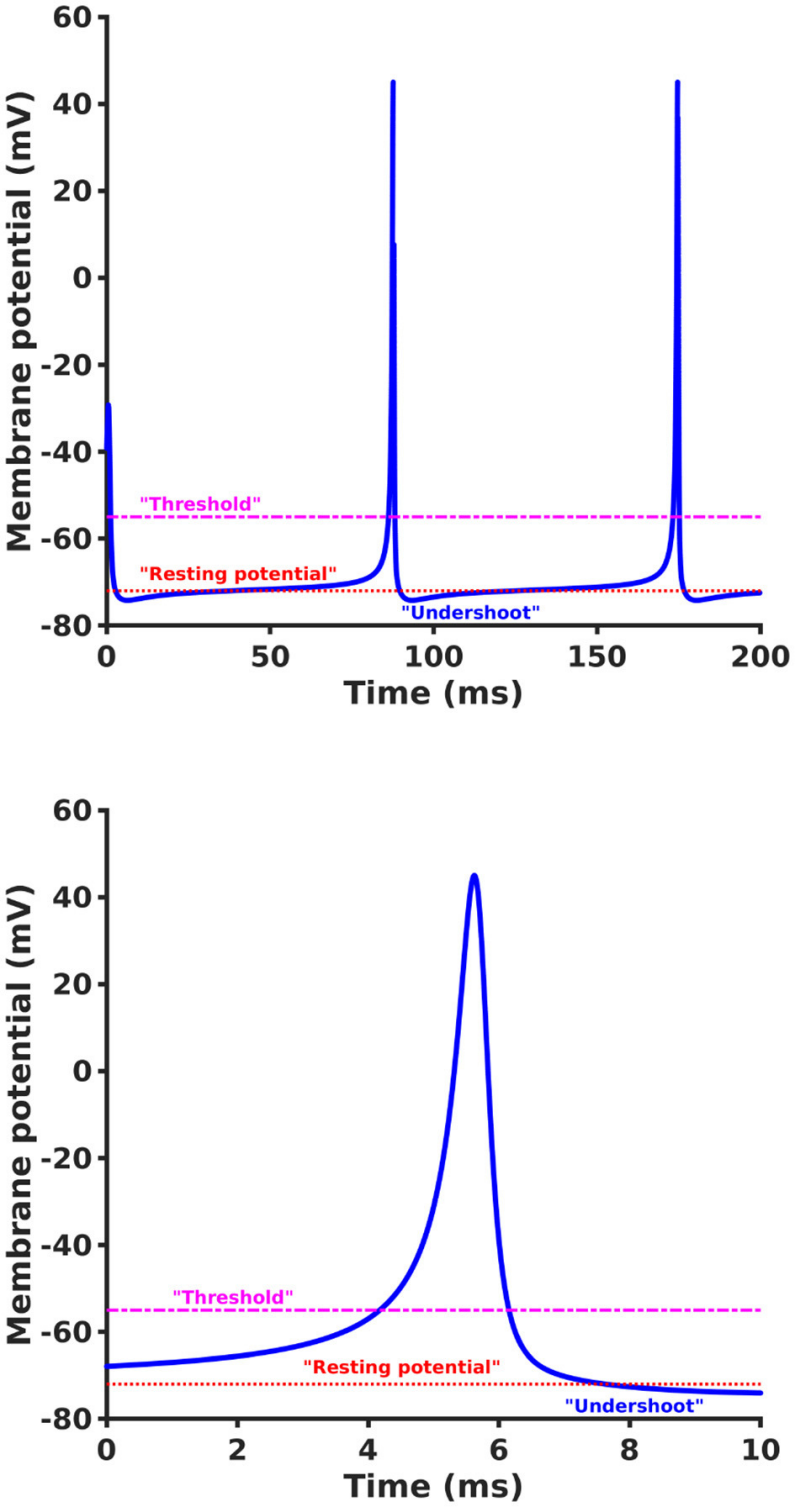
An example of a numerical solution of Equation 39 showing non-linear evolution of *a*_*k*_(*t*) as a function of *t* **(top)** and an expanded view of a single spike **(bottom)** using $\beta_{k}^{\prime }=\exp\left(i\pi /4\right)$, β_*k*_ = 2 exp(−*i*π/4), α_*k*_ = 3, and γ_*k*_ = 1.996 + *i*. The solution shows behavior in close agreement with typical axonal spiking but is derived directly from first principles of electrodynamics and wave propagation without any reference to the standard *ad hoc* reaction-diffusion approach of HH.

This solution to the non-linear problem can be directly applied to the case of the single axon, using experimentally measured physiological parameters, thus providing a more precise characterization of the propagating action potential. An example of a numerical solution of Equation 39 for the non-resonant condition using $\beta_{k}^{\prime }=\exp\left(i\pi /4\right)$, β_*k*_ = 2 exp(−*iπ*/4), α_*k*_ = 3, and γ_*k*_ = 1.996 + *i* is shown in Figure 2. The solution shows behavior in close agreement with a typical axonal action potential.

We emphasize that this result was derived from first principles based on the electrical properties of the axon without the need for an artificial reaction-diffusion model with multiple adjustable parameters (thresholds, time constants, etc.). In particular, Equation 39 reveals that the inverse proportionality of frequency and wave number in the brain wave dispersion relation admits a closed analytical form of a wave non-linear equation whose solution is a persistent traveling axonal non-linear wave (i.e., the action potential spike) resulting from the collective non-resonant interactions of multiple low amplitude wave modes.

We note here that, although we have assumed an idealized, perfectly cylindrical model for clarity, the same formalism can be extended to more complex geometries. However, it should also be recognized that the propagating non-linear electrodynamic waves have the capability of deforming the geometry of the charged membrane, which is consistent with theoretical as discussed in the study by El Hady and Machta (2015) and observational as discussed in the study by Ling et al. (2018) evidence of mechanistic waves (APPulse; Johnson and Winlow, 2018b,a) that accompany the action potential propagation.

### 2.10 Critical behavior of waves

#### 2.10.1 Critical points

We have previously demonstrated in previous studies (Galinsky and Frank, 2021c, 2023a,c) that Equation 39 can be rewritten in terms of a pair of coupled equations for the amplitude and phase as


(40) $$\begin{array}{l} \frac{dA}{dt}=\gamma A+A^{2}\left[R_{a}\cos\left(\phi -\Phi \right)-\alpha \right], \end{array}$$

(41) $$\begin{array}{l} \frac{d\phi }{dt}=\omega +AR_{\phi }\cos\phi , \end{array}$$

where we omitted the subscript *k* from all variables and assumed *a*(*t*) = *A*(*t*)*e*^*iϕ*(*t*)^. The parameters *R*_*a*_, *R*_ϕ_, and Φ can be expressed through β, and β′ as shown in the previous studies (Galinsky and Frank, 2021c, 2023a,c).

An equilibrium (i.e., *dA*/*dt* = *dϕ*/*dt* = 0) solution of Equations 37, 39 can be found from


(42) $$\begin{array}{l} -\frac{\gamma }{\omega }R_{\phi }\cos\phi +R_{a}\cos\left(\phi -\Phi \right)-\alpha =0, \end{array}$$

with equilibrium values ϕ_*e*_ ≡ const and *A*_*e*_ = −ω/*R*_ϕ_ cos ϕ_*e*_ = −γ/(*R*_*a*_ cos(ϕ_*e*_ − Φ) − α) ≡ const. This shows that for α > *R*_*a*_|sin Φ| there exist critical values *A*_*c*_, ϕ_*c*_, and μ_*c*_ (μ = γ/ω) where the equilibrium solution vanishes, such that


(43) $$\begin{array}{l} \mu_{c}=\frac{R_{a}\cos\left(\phi_{c}-\Phi \right)-\alpha }{R_{\phi }\cos\phi_{c}} \end{array}$$

(44) $$\begin{array}{l} \;=\frac{1}{R_{\phi }}\left[R_{a}\cos\Phi \pm \sqrt{\alpha^{2}-{\left(R_{a}\sin\Phi \right)}^{2}}\right] \end{array}$$

(45) $$\begin{array}{l} \phi_{c}=\arctan\left[\frac{R_{a}\sin\Phi }{R_{a}\cos\Phi -\mu_{c}R_{\phi }}\right] \end{array}$$

(46) $$\begin{array}{l} \;=\arctan\left[\frac{R_{a}\sin\Phi }{\pm \sqrt{\alpha^{2}-{\left(R_{a}\sin\Phi \right)}^{2}}}\right], \end{array}$$

(47) $$\begin{array}{l} A_{c}=-\frac{\omega }{R_{\phi }\cos\phi_{c}}=-\frac{\gamma }{\mu_{c}R_{\phi }\cos\phi_{c}}. \end{array}$$

These solutions provide the basis for an analysis of the critical regimes via a bifurcation analysis.

#### 2.10.2 Bifurcation analysis

The standard approach to analyzing the behavior of critical systems is to linearize the system equations around the critical point, then determine the stability of the system via the eigenvalues of the Jacobian (e.g., Strogatz, 2000). The linearized system of Equations 37, 39 at the critical point (*A*_*c*_, ϕ_*c*_) results in


(48) $$\begin{array}{l} \frac{dA}{dt}=\left(\gamma +2A_{c}\left[R_{a}\cos\left(\phi_{c}-\Phi \right)-\alpha \right]\right)A \end{array}$$

(49) $$\begin{array}{l} \;-A_{c}^{2}R_{a}\sin\left(\phi_{c}-\Phi \right)\phi , \end{array}$$

(50) $$\begin{array}{l} \frac{d\phi }{dt}=R_{\phi }\cos\left(\phi_{c}\right)A-A_{c}R_{\phi }\sin\left(\phi_{c}\right)\phi . \end{array}$$

For different parameter ranges, the system (see Equations 44, 45) [and hence the original system (Equations 37, 39 or 36)] shows different behavior corresponding to different bifurcation types, including both the saddle node on an invariant circle (SNIC) bifurcation (representative for Type I axon spiking) and Hopfbifurcation (that is claimed to be responsible for Type II axon spiking) (Prescott, 2014). For example, taking a limiting case of *R*_*a*_ ~ α with Φ = 0 (or Φ = π) and ϕ_*c*_ = π, the eigenvalues of the Jacobian matrix become


(51) $$\begin{array}{l} \lambda_{1}=0,\;\lambda_{2}=\gamma -2\omega \frac{\alpha \pm R_{a}}{R_{\phi }}, \end{array}$$

thus the system undergoes the SNIC bifurcation (λ_1_ = 0 and λ_2_ < 0 for for μ < 2μ_*c*_).

For an alternative limiting case of *R*_*a*_ ≪ α with Φ = −π/2 and ϕ_*c*_ ≈ π, the eigenvalues of the Jacobian matrix become


(52) $$\begin{array}{l} \lambda_{1,2}=q\pm \sqrt{q^{2}-\omega^{2}R_{a}/R_{\phi }} \end{array}$$

(53) $$\begin{array}{l} q=\frac{\gamma }{2}-\omega \frac{\alpha }{R_{\phi }} \end{array}$$

and in this case for *q* = 0 (or μ = 2μ_*c*_), the eigenvalues λ_1, 2_ are pure imaginary, crossing the imaginary axis with a change of parameter μ (either ω or γ), which is an example of a Hopf bifurcation. Thus, the wave model of action potential shows that the non-linear axon wave includes multiple critical regimes and produces different spiking behavior consistent with different experimentally detected types.

It should be noted that the non-linear system (Equations 37, 39) is not a simple harmonic oscillator system. For a harmonic oscillator the amplitude *A* is constant (does not change at all) and the phase ϕ is changing rapidly with a constant rate ω. The non-linear system (Equations 37, 39) in the subcritical regime, that is, when μ < μ_*c*_, shows the oscillations where the rate of phase change is not constant anymore and the amplitude *A* is changing as well, reaching the maximum *A*_*max*_ = γ/(α − *R*_*a*_) and the minimum *A*_*min*_ = γ/(α + *R*_*a*_) for *dA*/*dt* = 0 when ϕ = Φ and ϕ = Φ + π respectively.

#### 2.10.3 Spike rate analysis

As discussed in the studies by Galinsky and Frank (2021c, 2023a,c), we can estimate the effective period of spiking *T*_*s*_ (or its inverse—either the firing rate 1/*T*_*s*_ or the effective firing frequency 2π/*T*_*s*_) from Equation 39 by substituting *A* with either *A*_*min*_ (for positive spikes, |ϕ_*c*_ − Φ| > π/2) or *A*_*max*_ (for negative spikes, |ϕ_*c*_ − Φ| < π/2) as for the most of the time (except for the short spike duration time), the amplitude *A* will be close to one of those values, hence


(54) $$\begin{array}{l} T_{s}=\overset{2\pi }{\underset{0}{\int }}\frac{d\phi }{\omega +\frac{\gamma R_{\phi }}{\alpha \pm R_{a}}\cos\phi }=\frac{1}{\omega }\overset{2\pi }{\underset{0}{\int }}\frac{d\phi }{1+\frac{\mu }{\mu_{c}}\nu \cos\phi } \end{array}$$

(55) $$\begin{array}{l} \;=\frac{2\pi }{\omega \sqrt{1-\nu^{2}\mu^{2}/\mu_{c}^{2}}}, \end{array}$$

where


(56) $$\begin{array}{l} \nu =\frac{R_{a}\cos\left(\phi_{c}-\Phi \right)-\alpha }{\left(\alpha \pm R_{a}\right)\cos\phi_{c}}, \end{array}$$

and the effective firing frequency ω_*s*_


(57) $$\begin{array}{l} \omega_{s}=\frac{2\pi }{T_{s}}=\omega \sqrt{1-\nu^{2}\mu^{2}/\mu_{c}^{2}}. \end{array}$$

As in the case discussed above where Φ = 0 (or Φ = π) and ϕ_*c*_ = π (also addressed in the studies by Galinsky and Frank, 2021c, 2023a,c) results in ν = 1, hence gives ω_*s*_ = 0 when μ reaches the critical value μ_*c*_, that is it allows spiking with arbitrary low frequencies—the typical behavior of Type I neurons (Prescott, 2014). In the alternative case of Φ = −π/2 and ϕ_*c*_ = π, ν = α/(α + *R*_*a*_) < 1, hence at the critical point the spiking frequency ω_*s*_ cannot be less than the minimum value of $\omega \sqrt{1-\nu^{2}}>0$—the behavior attributed to Type II neurons (Prescott, 2014).

#### 2.10.4 Influence of the applied potential

Our construction of the conductivity tensor defined in Equation 14 included an adjustable normalization *V* that represents the equilibrium voltage drop across the membrane as the vast majority of experiments investigating neuronal spiking involve some form of manipulation of *V*, such as “voltage clamping.” From the dispersion relation expressions (Equations 19–22, 26) it follows that


(58) $$\begin{array}{l} \mu =\frac{\gamma_{e}-\gamma_{d}}{\omega }=\mu_{0}+\frac{\gamma_{d}}{\omega_{0}}\left(1-\frac{V_{0}}{V}\right), \end{array}$$

where μ_0_ and ω_0_ are the critical parameters and the linear wave frequency evaluated at *V* = *V*_0_. Therefore, in the subcritical (μ < μ_*c*_) regime increasing the voltage difference *V* across the membrane, or hyperpolarizing the membrane, increases the criticality parameter μ, hence decreases the firing frequency ω_*s*_, stopping the oscillatory (spiking) behavior completely when the critical point μ_*c*_ is reached. In the super-critical (μ > μ_*c*_) case, that is, when the neuron is not firing, decreasing the voltage difference *V* (depolarizing the membrane as it is done in voltage clamping experiments) decreases the criticality parameter μ and makes neuron fire either at non-zero frequency (similar to Type II neuron) or at arbitrary low frequency (similar to Type I neuron). A special case of a neuron firing a single spike at the critical point may also appear if an update of the cross-membrane voltage proceeds too slowly and the system is able to relax back and stay at or above the critical point. Still, the periodic firing will emerge with increasing firing frequency ω_*s*_ when depolarization continues moving μ further in the subcritical range.

#### 2.10.5 Implications for neural networks

As shown in Galinsky and Frank (2021a,b, 2023b), the network formed from such non-linear oscillators exhibits synchronization properties that neither linear oscillators nor diffusive-reactive HH neurons can produce. Therefore, the current view that a single neuron can be approximated by the reactive “point neuron” HH system, which communicates through cable-like diffusive signal propagation with other neurons in networks of interconnected neurons, may not be entirely appropriate for understanding the dynamics of brain communication. A more appropriate view may be to consider that the critical synchronized state is formed both at a single neuron level and in their interconnected networks by multiple waves that are constantly generated at axonal membranes, interact and propagate along those membrane interfaces, making the networks they form to be more appropriately analogous to webs of highly tensioned strings rather than networks of leaky pipes with slow diffusive flow of some substance inside those pipes.

In this “string theory” view of neural networks, the specific details of the complex biochemical processes that mediate membrane voltages are not seen as the actual mechanism behind axonal spiking nor the subsequent signal propagation in single neurons and networks of neurons. Instead, the details about opening and closing of voltage-gated channels, about different number of Na^+^, K^+^, Cl^−^, Ca^2+^, etc., channels, about differences in kinetics of those carrier channels, about operation of ATP mediated carrier pumps, etc., all serve to “tune” the membranal strings by keeping the individual membranes, and hence, the network as a whole, at or close to the critical level.

## 3 Conclusion

Highly non-linear systems in nature present a significant problem in data analysis and interpretation because they can produce a wide variety of seemingly disparate and unrelated coherent phenomena. This is particularly true in critical systems, where small parameter variations produce drastically different system configurations. Without a physical model for such systems, one is left with a confusing conglomeration of experimentally observed and often seemingly contradictory effects without a guiding principle for understanding the underlying system dynamics. Additionally, without a guiding theoretical framework, data analysis strategies usually rely on essentially *ad hoc* fitting methods. The more complex the system, the more parameters are required. Such strategies make it possible to fit the data, but deriving a link to the actual system dynamics in the absence of a theoretical framework is problematic.

The human brain is a spectacular example of such a non-linear critical system. However, the lack of a physical theory of brain activity has led research down that familiar pathway. So, while the pioneering study of Hodgkin and Huxley (1952) provided a new unifying framework for fitting the action potential, it must be recognized for what it is: an *ad hoc* multiparameteric fitting method without a physical model. It is not surprising, then, that it has some glaring weaknesses, as noted above, not least of which is the difficulty in relating the neuronal action potential to large-scale brain network communication. Nevertheless, it has remained the standard model for the action potential. It forms the basis for subsequent methods that rely on the empirical fitting of a single measured axonal signal waveform to a set of *ad hoc* multi-parametric differential equations with multiple fitting parameters as is typically employed by a multitude of single-neuron spiking models (Fitzhugh, 1961; Nagumo et al., 1962; Morris and Lecar, 1981; Izhikevich, 2003; Gerstner et al., 2014; Kulkarni et al., 2020; Kim and Sejnowski, 2021).

Our intention in writing this article was not to develop an alternative model that allows for simply fitting a signal to any biological/neuronal experiment—there is little doubt that a model with a couple of dozen adjustable parameters is well-suited for this purpose. Indeed, this is what the standard HH model provides. Instead, the focus of our study directed at showing that a straightforward application of fundamental physical principles can be used to demonstrate that membrane non-linearity is not restricted to radial effects only, but also produces axial terms that are ignored by HH consideration, thus allowing us to obtain the same action potential with properties that naturally follow from a wave model that does not require a multitude of parameters. A key finding of the study is that this relatively simple wave description follows directly from our more general theory, as developed in our previous publications.

Our recent development of a general physical model (WETCOW) for brain activity derived from the first principles of electrodynamics Galinsky and Frank (2020b,a, 2021a,b) was motivated by the desire to address this problem by constructing a single unifying framework for understanding brain activity at all scales, from neuron to network. Subsequent studies focused on the large-scale effects such as network synchronization, learning, and neuronal avalanches Galinsky and Frank (2023b,a,c). While this model was developed with all neural tissues in mind, and is therefore implicitly applicable to single neurons, we never explicitly addressed this problem, which involved applying the general theory to the specific tissue model of a single neuron. The objective of this study was to address this problem and thereby explicitly demonstrate that our general theory is applicable at the range of scales relevant to brain activity.

In doing so, we have demonstrated that this theory of the neuron action potential is the same that has already demonstrated the ability to explain multiple observed macroscopic brain electric activity, such as extracellular spiking, efficient brain synchronization, neuronal avalanches, and memory and learning mechanisms (Galinsky and Frank, 2020b,a, 2021a, 2023b,a,c) that are not explained by the standard HH model. We have thus demonstrated a theory that bridges the gap between the most elemental brain electrical unit—the neuron—and the large-scale, collective, synchronous behavior of the brain.

The construction of a physical theory from the first principles of electrodynamics begged the question of the relationship to existing electrodynamic models. The most obvious candidate is the ubiquitous “cable theory,” which has a long history in attempts to describe neuronal signals. However, as we demonstrated in Section 2.4, it is derivable from our more general theory, but only by imposing conditions that limit its applicability to real neurons. The cable equations were subsequently shown to be inadequate to characterize the action potential under a wide range of realistic conditions.

Recognition that the HH model has never been capable of solving the problem of characterizing the action potential in myelinated axons led us to consider that problem within our theory. We found that the solution was straightforward because our theory explicitly incorporates both geometrical and physiological tissue parameters. This resulted in predictions for wave speeds consistent with measured values in both myelinated and unmyelinated axons. It is worth noting that these results have practical significance because they provide a direct method for relating neuronal activity to disease states characterized by demyelination, such as Multiple Sclerosis (Coutinho Costa et al., 2023), and myelin pathogenesis, including Alzheimer's Disease (Cai and Xiao, 2016; Maitre et al., 2023).

Our wave model does not specify the direction of wave travel, suggesting possible reflection at axonal ends. Biological networks, however, exhibit preferred signal directions. The amount of reflection at the axonal ends depends on the exact geometry and boundary conditions in the axial coordinate. We did not address the question of reflection in the study, as it falls beyond the main focus of the study. Nevertheless, the typical geometry of axon shows decrease of the diameter from proximal to distal areas that would result in decrease of speed and increase of linear wave amplitude for proximal to distal propagation, and increase of speed and reduction of linear wave amplitude for opposite direction of propagation, meaning that even without choosing specific initial and boundary conditions the reflection of wave will not be favored.

The ability of our general WETCOW theory to describe both spatially extended (including network-level) effects as well as neuron-scale effects led to the demonstration of some remarkable similarities between the two scales of brain phenomena. In Section 2.10 we demonstrated that the critical behavior previously shown to be evident in collective synchronous spiking and neuronal avalanches (Galinsky and Frank, 2021c, 2023a,c), was similarly manifest in the neuronal signal where now it corresponds to the characteristics of Type I and Type II neurons. The modeling of synaptic interactions within this framework would be done conceptually similar to network models presented in our previous works (Galinsky and Frank, 2021a,b, 2023b).

One obvious question these results raise is the logic of the current view of neuronal signaling being *created* by the HH mechanism of ion exchange, particularly in light of the demonstrated inadequacy of the diffusion picture. The traveling coherent non-linear waves predicted by our theory, based solely on the bioelectric properties of the tissues, will cause a time-dependent voltage drop across the neuronal membrane, influencing transmembrane permeability and thereby allowing the opening and closing of multiple voltage-gated channels in synchrony. In this view, the problematic question of how ion channels mysteriously synchronize to produce an action potential does not arise.

## Data Availability

The original contributions presented in the study are included in the article/Supplementary material, further inquiries can be directed to the corresponding author.
