# Supplementary material for: The wave nature of the action potential
Source: Front Cell Neurosci. 2025 Apr 25;19:1467466. doi: 10.3389/fncel.2025.1467466 (PMC12062021; doi:10.3389/fncel.2025.1467466)
Supplement: Supplementary file 1 [file Supplementary_file_1.pdf]

frontiers

Supplementary Material

Appendices

1 CHARGE CONTINUITY EQUATION

Starting with the usual expression for the Ampère's law:

$$\nabla \times \mathbf{H}(\mathbf{r},t) = \mathbf{J}(\mathbf{r},t) + \frac{\partial \mathbf{D}(\mathbf{r},t)}{\partial t} \tag{A1}$$

Taking the divergence yields:

$$0 = \nabla \cdot \mathbf{J}(\mathbf{r},t) + \frac{\partial \nabla \cdot \mathbf{D}(\mathbf{r},t)}{\partial t} \tag{A2}$$

Assuming the (inhomogeneous, linear and non-dispersive) constitutive relations:

$$\mathbf{D}(\mathbf{r},t) = \epsilon(\mathbf{r})\mathbf{E}(\mathbf{r},t) \tag{A3}$$

and

$$\mathbf{J}(\mathbf{r},t) = \mathbf{J}_s(\mathbf{r},t) + \boldsymbol{\sigma}(\mathbf{r}) \cdot \mathbf{E}(\mathbf{r},t) \tag{A4}$$

where $\epsilon(\mathbf{r})$ is a scalar and $\boldsymbol{\sigma}(\mathbf{r})$ is a tensor. The quantity $\mathbf{J}_s(\mathbf{r},t)$ represents an impressed source current.

Substituting these relations into equation (A2) above yields:

$$-\nabla \cdot \mathbf{J}_s(\mathbf{r},t) = \nabla \cdot (\boldsymbol{\sigma}(\mathbf{r}) \cdot \mathbf{E}(\mathbf{r},t)) + \frac{\partial(\nabla \cdot (\epsilon(\mathbf{r})\mathbf{E}(\mathbf{r},t)))}{\partial t} \tag{A5}$$

Multiplying equation (A5) by $\epsilon(\mathbf{r})$, defining the permittivity scaled conductivity tensor $\boldsymbol{\Sigma}(\mathbf{r}) = \frac{\sigma(\mathbf{r})}{\epsilon(\mathbf{r})}$, and expressing the electric field in the quasi-electrostatic approximation as $\mathbf{E}(\mathbf{r},t) = -\nabla\phi(\mathbf{r},t)$ produces:

$$\epsilon(\mathbf{r})\nabla \cdot \mathbf{J}_s(\mathbf{r},t) = \epsilon(\mathbf{r})\nabla \cdot (\boldsymbol{\Sigma}(\mathbf{r}) \cdot \epsilon(\mathbf{r})\nabla\phi(\mathbf{r},t)) + \frac{\partial(\epsilon(\mathbf{r})\nabla \cdot (\epsilon(\mathbf{r})\nabla\phi(\mathbf{r},t)))}{\partial t} \tag{A6}$$

Rewriting equation (A6) in component form, assuming Cartesian coordinates and ignoring the source term:

$$0 = \epsilon(\mathbf{r})\frac{\partial}{\partial x_i}\left(\Sigma_{ij}(\mathbf{r})\epsilon(\mathbf{r})\frac{\partial}{\partial x_j}\phi(\mathbf{r},t)\right) + \frac{\partial}{\partial t}\left(\epsilon(\mathbf{r})\frac{\partial}{\partial x_i}\left(\epsilon(\mathbf{r})\frac{\partial}{\partial x_i}\phi(\mathbf{r},t)\right)\right) \tag{A7}$$

where summation over repeated indices is assumed.

Introducing coordinate transform vectors $(\mathbf{u}, \mathbf{v}, \mathbf{w})$ of a new coordinate system (u, v, w) can be expressed using vectors $(\mathbf{x}, \mathbf{y}, \mathbf{z})$ of an original global Cartesian coordinate system (x, y, z) as

$$\mathbf{u} = \frac{\partial x}{\partial u}\mathbf{x} + \frac{\partial y}{\partial u}\mathbf{y} + \frac{\partial z}{\partial u}\mathbf{z}$$
$$\mathbf{v} = \frac{\partial x}{\partial v}\mathbf{x} + \frac{\partial y}{\partial v}\mathbf{y} + \frac{\partial z}{\partial v}\mathbf{z}$$
$$\mathbf{w} = \frac{\partial x}{\partial w}\mathbf{x} + \frac{\partial y}{\partial w}\mathbf{y} + \frac{\partial z}{\partial w}\mathbf{z} \tag{A8}$$

The differential element $d\mathbf{r}$ in this transformed coordinates system can be written as

$$d\mathbf{r} = \frac{\partial \mathbf{r}}{\partial u}du + \frac{\partial \mathbf{r}}{\partial v}dv + \frac{\partial \mathbf{r}}{\partial w}dw = h_u du\mathbf{u} + h_v dv\mathbf{v} + h_w dw\mathbf{w}, \tag{A9}$$

where (h_u, h_w, h_w) are the scale factors. Using the rotated global Cartesian coordinate system they can be expressed only through diagonal elements

$$h_u = \left|\frac{\partial x}{\partial u}\right|, \quad h_v = \left|\frac{\partial y}{\partial v}\right|, \quad h_w = \left|\frac{\partial z}{\partial w}\right|, \tag{A10}$$

for the original global Cartesian coordinate system they are defined as a projection of the differential element $d\mathbf{r}$

$$h_u = \left|\frac{\partial \mathbf{r}}{\partial u}\right|, \quad h_v = \left|\frac{\partial \mathbf{r}}{\partial v}\right|, \quad h_w = \left|\frac{\partial \mathbf{r}}{\partial w}\right|. \tag{A11}$$

Irrespective of their definition, for reasonably “good” permittivity (i.e., meaning that ϵ is strictly positive and bounded anywhere inside the domain of interest) we can always construct our transformation such that

$$h_u = h_v = h_w = 1/\epsilon(x(u, v, w), y(u, v, w), z(u, v, w)). \tag{A12}$$

It is well known that for the gradient of any arbitrary scalar function ϕ we can write that

$$\nabla\phi = \frac{1}{h_u}\frac{\partial \phi}{\partial u}\mathbf{u} + \frac{1}{h_v}\frac{\partial \phi}{\partial v}\mathbf{v} + \frac{1}{h_w}\frac{\partial \phi}{\partial w}\mathbf{w}. \tag{A13}$$

And for the divergence of any arbitrary vector function $\mathbf{S}$ we can write that

$$\nabla \cdot \mathbf{S} = \frac{1}{h_u h_v h_w}\left(\frac{\partial h_v h_w S_u}{\partial u} + \frac{\partial h_u h_w S_v}{\partial v} + \frac{\partial h_u h_v S_w}{\partial w}\right). \tag{A14}$$

Applying the coordinate transformation to all terms in equation (A7) we get

$$0 = \frac{\epsilon}{h_u h_v h_w}\Bigg\{\Bigg[\Bigg[\frac{\partial}{\partial u}\epsilon\left(\mathbf{\Sigma}_{uu}h_v h_w\frac{1}{h_u}\frac{\partial \phi}{\partial u} + \mathbf{\Sigma}_{uv}h_u h_w\frac{1}{h_v}\frac{\partial \phi}{\partial v} + \mathbf{\Sigma}_{uw}h_u h_v\frac{1}{h_w}\frac{\partial \phi}{\partial w}\right)\Bigg] +$$

$$\left[\frac{\partial}{\partial v}\epsilon\left(\boldsymbol{\Sigma}_{vu}h_v h_w\frac{1}{h_u}\frac{\partial\phi}{\partial u}+\boldsymbol{\Sigma}_{vv}h_u h_w\frac{1}{h_v}\frac{\partial\phi}{\partial v}+\boldsymbol{\Sigma}_{vw}h_u h_v\frac{1}{h_w}\frac{\partial\phi}{\partial w}\right)\right]+$$

$$\left.\left[\frac{\partial}{\partial w}\epsilon\left(\boldsymbol{\Sigma}_{wu}h_v h_w\frac{1}{h_u}\frac{\partial\phi}{\partial u}+\boldsymbol{\Sigma}_{wv}h_u h_w\frac{1}{h_v}\frac{\partial\phi}{\partial v}+\boldsymbol{\Sigma}_{ww}h_u h_v\frac{1}{h_w}\frac{\partial\phi}{\partial w}\right)\right]\right]+$$

$$\left.\frac{\partial}{\partial t}\left[\left[\frac{\partial}{\partial u}\epsilon h_v h_w\frac{1}{h_u}\frac{\partial\phi}{\partial u}+\frac{\partial}{\partial v}\epsilon h_u h_w\frac{1}{h_v}\frac{\partial\phi}{\partial v}+\frac{\partial}{\partial w}\epsilon h_u h_v\frac{1}{h_w}\frac{\partial\phi}{\partial w}\right]\right]\right\}, \tag{A15}$$

As we have $h_u = h_v = h_w = 1/\epsilon$ after canceling the common factor $\epsilon/(h_u h_v h_w)$ we obtain

$$0=\left\{\left[\left[\frac{\partial}{\partial u}\left(\Sigma_{uu}\frac{\partial\phi}{\partial u}+\Sigma_{uv}\frac{\partial\phi}{\partial v}+\Sigma_{uw}\frac{\partial\phi}{\partial w}\right)\right]+\right.\right.$$

$$\left[\frac{\partial}{\partial v}\left(\Sigma_{vu}\frac{\partial\phi}{\partial u}+\Sigma_{vv}\frac{\partial\phi}{\partial v}+\Sigma_{vw}\frac{\partial\phi}{\partial w}\right)\right]+$$

$$\left.\left[\frac{\partial}{\partial w}\left(\Sigma_{wu}\frac{\partial\phi}{\partial u}+\Sigma_{wv}\frac{\partial\phi}{\partial v}+\Sigma_{ww}\frac{\partial\phi}{\partial w}\right)\right]\right]+$$

$$\left.\frac{\partial}{\partial t}\left[\left[\frac{\partial^2\phi}{\partial u^2}+\frac{\partial^2\phi}{\partial v^2}+\frac{\partial^2\phi}{\partial w^2}\right]\right]\right\}, \tag{A16}$$

or in primed notation $((u,v,w)->(x'))$

$$0=\frac{\partial}{\partial x'_i}\left(\boldsymbol{\Sigma}_{ij}(\mathbf{r}')\frac{\partial}{\partial x'_j}\phi(\mathbf{r}',t)\right)+\frac{\partial}{\partial t}\left(\frac{\partial}{\partial x'_i}\left(\frac{\partial}{\partial x'_i}\phi(\mathbf{r}',t)\right)\right). \tag{A17}$$

i.e., the equation (3) of the section section 2.1 that is valid for any general form of permittivity ϵ.

2 LINEAR SYSTEM

Defining

$$\boldsymbol{u}\equiv\begin{pmatrix}\frac{1}{r}\frac{\partial}{\partial r}r\\ \frac{1}{r}\frac{\partial}{\partial\theta}\\ \frac{\partial}{\partial z}\end{pmatrix},\ \boldsymbol{\Sigma}(\boldsymbol{r})\equiv\begin{pmatrix}\Sigma_{rr}(r,\theta,z) & \Sigma_{r\theta}(r,\theta,z) & \Sigma_{rz}(r,\theta,z)\\ \Sigma_{\theta r}(r,\theta,z) & \Sigma_{\theta\theta}(r,\theta,z) & \Sigma_{\theta z}(r,\theta,z)\\ \Sigma_{zr}(r,\theta,z) & \Sigma_{z\theta}(r,\theta,z) & \Sigma_{zz}(r,\theta,z)\end{pmatrix},\ \boldsymbol{v}\equiv\begin{pmatrix}\frac{\partial\phi}{\partial r}\\ \frac{1}{r}\frac{\partial\phi}{\partial\theta}\\ \frac{\partial\phi}{\partial z}\end{pmatrix} \tag{A18}$$

equation (A17) can be written in cylindrical (r,θ,z) coordinate system as

$$\frac{\partial}{\partial t}\left(\boldsymbol{u}^t\cdot\boldsymbol{v}\right)=-\boldsymbol{u}^t\cdot\boldsymbol{\Sigma}\cdot\boldsymbol{v} \tag{A19}$$

or

$$\frac{\partial}{\partial t}\left(\frac{1}{r}\frac{\partial}{\partial r}r\frac{\partial\phi}{\partial r}+\frac{1}{r^2}\frac{\partial^2\phi}{\partial\theta^2}+\frac{\partial^2\phi}{\partial z^2}\right)=-\frac{1}{r}\frac{\partial}{\partial r}r\left(\Sigma_{rr}\frac{\partial\phi}{\partial r}+\Sigma_{r\theta}\frac{1}{r}\frac{\partial\phi}{\partial\theta}+\Sigma_{rz}\frac{\partial\phi}{\partial z}\right)$$

$$-\frac{1}{r}\frac{\partial}{\partial\theta}\left(\Sigma_{\theta r}\frac{\partial\phi}{\partial r}+\Sigma_{\theta\theta}\frac{1}{r}\frac{\partial\phi}{\partial\theta}+\Sigma_{\theta z}\frac{\partial\phi}{\partial z}\right)-\frac{\partial}{\partial z}\left(\Sigma_{zr}\frac{\partial\phi}{\partial r}+\Sigma_{z\theta}\frac{1}{r}\frac{\partial\phi}{\partial\theta}+\Sigma_{zz}\frac{\partial\phi}{\partial z}\right) \tag{A20}$$

Assuming that in linear regime the conductivity tensor Σ^0 inside the membrane ($1 \le r \le 1+\bar{\delta}, 0 \le \theta < 2\pi, -\infty < z < \infty$) is diagonal and position independent

$$\mathbf{\Sigma^0} \equiv \begin{pmatrix} \Sigma^0_{rr} & 0 & 0 \\ 0 & \Sigma^0_{\theta\theta} & 0 \\ 0 & 0 & \Sigma^0_{zz} \end{pmatrix}, \tag{A21}$$

equation (A20) becomes

$$\frac{\partial}{\partial t}\left(\frac{1}{r}\frac{\partial}{\partial r}r\frac{\partial\phi}{\partial r}+\frac{1}{r^2}\frac{\partial^2\phi}{\partial\theta^2}+\frac{\partial^2\phi}{\partial z^2}\right)=-\Sigma^0_{rr}\frac{1}{r}\frac{\partial}{\partial r}r\frac{\partial\phi}{\partial r}-\Sigma^0_{\theta\theta}\frac{1}{r^2}\frac{\partial^2\phi}{\partial\theta^2}-\Sigma^0_{zz}\frac{\partial^2\phi}{\partial z^2} \tag{A22}$$

3 SOLUTION OF LINEAR SYSTEM

3.1 Particular solution with inhomogeneous boundary conditions

Inhomogeneous boundary conditions for the membrane domain are

$$\begin{aligned} &\phi(r,\theta,z)|_{r=1}=0, \qquad \phi(r,\theta,z)|_{r=1+\bar{\delta}}=1, \\ &\phi(r,\theta,z)|_{\theta=0}=\phi(r,\theta,z)|_{\theta=2\pi}, \\ &\phi(r,\theta,z)|_{z=-\infty}=\phi(r,\theta,z)|_{z=\infty} \end{aligned} \tag{A23}$$

The particular solution of (A22) then requires

$$\frac{1}{r}\frac{d}{dr}\left(r\frac{d\phi^0}{dr}\right)=0 \Longrightarrow r\frac{d\phi^0}{dr}= \text{ constant }, \Longrightarrow \phi^0(r)=\frac{\ln r}{\ln(1+\bar{\delta})}\approx\frac{1}{\bar{\delta}}\ln r, \tag{A24}$$

3.2 Eigen solutions of the linear system with homogeneous boundary conditions

Introducing the homogeneous eigenvalue problem as

$$\frac{1}{r}\frac{\partial}{\partial r}r\frac{\partial\phi}{\partial r}+\frac{1}{r^2}\frac{\partial^2\phi}{\partial\theta^2}+\frac{\partial^2\phi}{\partial z^2}=-\varkappa^2\phi, \tag{A25}$$

the solution of linear equation (A22) is separable in radial, axial and azimuthal directions ($\phi(t,r,\theta,z)=\phi_{\kappa kn}(t)=\phi_t(t)\phi_k(z)\phi_n(\theta)\phi_{\kappa_n}(r)$), such that they satisfy

$$\frac{d^2\phi_k}{dz^2}=-k^2\phi_k, \tag{A26}$$

$$\frac{d^2\phi_n}{d\theta^2}=-n^2\phi_n, \tag{A27}$$

$$\frac{1}{r}\frac{d}{dr}r\frac{d\phi_{\kappa_n}}{dr}-\frac{n^2}{r^2}\phi_{\kappa_n}=-\kappa_n^2\phi_{\kappa_n}, \tag{A28}$$

where $\varkappa^2 = k^2 + \kappa_n^2$ with the solutions

$$\phi_{\kappa_n}(r) \sim R_n(\kappa_n r + \eta_n), \quad \phi_k(z) \sim e^{-ikz}, \quad \phi_n(\theta) \sim e^{-in\theta}, \tag{A29}$$

where R_n denotes Bessel functions either of the first (J_n) or the second (Y_n) kind, and κ_n and η_n can be determined from the boundary conditions, $R_n(\kappa_n + \eta_n) = R_n(\kappa_n(1+\bar{\delta}) + \eta_n) = 0$, so that the zeros of the Bessel function are located at $r = 1$ and $r = 1 + \bar{\delta}$. For $\bar{\delta} \ll 1$ the argument $x \equiv (\kappa_n r + \eta_n)$ of R_n is large, hence

$$J_n(x) \sim \sqrt{\frac{2}{\pi x}} \cos(x - \pi n/2 - \pi/4), \tag{A30}$$

$$Y_n(x) \sim \sqrt{\frac{2}{\pi x}} \sin(x - \pi n/2 - \pi/4), \tag{A31}$$

therefore, $\eta_n \sim \pi(2n \pm 1)/4$ and $\kappa_n \sim \pi m/\bar{\delta}$ where $m = 1 \dots \infty$. Alternatively, the R_n can be chosen as $R_n(\kappa_n r) = \alpha J_n(\kappa_n r) + \beta Y_n(\kappa_n r)$ with eigenvalues κ_n determined from the solvability condition $J_n(\kappa_n)Y_n(\kappa_n(1+\delta)) - J_n(\kappa_n(1+\delta))Y_n(\kappa_n) = 0$ for the homogeneous equation for α and β. In the large argument approximation this will result in the same expression for κ_n and in trivial expressions for α and β through η_n as $\alpha = C \cos\eta_n$ and $\beta = C \sin\eta_n$ ($C = \text{const}$).

Then from (A22)

$$\left(\kappa_n^2 + k^2\right) \frac{d\phi_{\kappa k n}(t)}{dt} = -\left[\kappa_n^2 \Sigma_{rr}^0 + k^2 \Sigma_{zz}^0 + \frac{n^2}{r^2}(-\Sigma_{rr}^0 + \Sigma_{\theta\theta}^0)\right] \phi_{\kappa k n}(t). \tag{A32}$$

After integrating (A32) over azimuthal θ dependence only terms with $n = 0$ will be left as $\int_0^{2\pi} \phi_n(\theta) d\theta = 0$ for any $n \neq 0$, hence for analysis of axial variations it is sufficient to use azimuthally averaged eigenmodes $\phi_{\kappa k 0}$ with $\kappa_0 \equiv \kappa$

$$\left(\kappa^2 + k^2\right) \frac{d\phi_{\kappa k 0}(t)}{dt} = -\left[\kappa^2 \Sigma_{rr}^0 + k^2 \Sigma_{zz}^0)\right] \phi_{\kappa k 0}(t), \tag{A33}$$

and as we show in section 2.4, the ubiquitous cable equation easily follows from the above linear model (A33).

4 NONLINEAR MEMBRANE RESPONSE

Starting for simplicity with equation (A17) (or (3)) in Cartesian coordinates assume that the conductivity tensor $\mathbf{\Sigma} \equiv \mathbf{\Sigma}(\Psi[\phi])$ has a linear functional dependence on ϕ (i.e., $(\mathbf{\Sigma}(\Psi[\phi_1 + \phi_2]) = \mathbf{\Sigma}(\Psi[\phi_1]) + \mathbf{\Sigma}(\Psi[\phi_2]))$, and that ϕ_0 is an equilibrium solution of (A17) with the conductivity tensor $\mathbf{\Sigma}^0 \equiv \mathbf{\Sigma}(\Psi[\phi^0])$. Assuming that the full solution ϕ is close to the equilibrium ϕ^0, defining $\phi = \phi^0 + \phi'$ (where ϕ' is a deviation from equilibrium) and $\mathbf{\Sigma}' \equiv \mathbf{\Sigma}'(\Psi[\phi - \phi^0])$, such that $\mathbf{\Sigma} = \mathbf{\Sigma}^0 + \mathbf{\Sigma}'$, an equation for ϕ' becomes

$$\frac{\partial}{\partial t} \nabla^2 \phi' = -\nabla \cdot \mathbf{\Sigma}^0 \cdot \nabla\phi' - \nabla \cdot \mathbf{\Sigma}' \cdot \nabla\phi^0 - \nabla \cdot \mathbf{\Sigma}' \cdot \nabla\phi'. \tag{A34}$$

Multiplying by $\phi' d\mathscr{V}$ and integrating by part in the entire domain $\mathscr{V}$

$$\frac{1}{2}\frac{\partial}{\partial t}\int\limits_{\mathscr{V}} |\nabla\phi'|^2 d\mathscr{V} = -\int\limits_{\mathscr{V}} \nabla\phi' \cdot \boldsymbol{\Sigma}^0 \cdot \nabla\phi' d\mathscr{V} - \int\limits_{\mathscr{V}} \nabla\phi' \cdot \boldsymbol{\Sigma}' \cdot \nabla\phi^0 d\mathscr{V} - \int\limits_{\mathscr{V}} \nabla\phi' \cdot \boldsymbol{\Sigma}' \cdot \nabla\phi' d\mathscr{V}. \quad \text{(A35)}$$

A deviation ϕ' is arbitrary therefore it can be set as $\phi = \phi^0 - \phi'$. In this case $\boldsymbol{\Sigma}'(\Psi[-\phi']) = -\boldsymbol{\Sigma}'(\Psi[\phi'])$ hence

$$\frac{1}{2}\frac{\partial}{\partial t}\int\limits_{\mathscr{V}} |\nabla\phi'|^2 d\mathscr{V} = -\int\limits_{\mathscr{V}} \nabla\phi' \cdot \boldsymbol{\Sigma}^0 \cdot \nabla\phi' d\mathscr{V} - \int\limits_{\mathscr{V}} \nabla\phi' \cdot \boldsymbol{\Sigma}' \cdot \nabla\phi^0 d\mathscr{V} + \int\limits_{\mathscr{V}} \nabla\phi' \cdot \boldsymbol{\Sigma}'] \cdot \nabla\phi' d\mathscr{V}. \quad \text{(A36)}$$

Both (A35) and (A36) can be valid only if for any arbitrary ϕ'

$$\int\limits_{\mathscr{V}} \nabla\phi' \cdot \boldsymbol{\Sigma}' \cdot \nabla\phi' d\mathscr{V} = 0 \quad \text{(A37)}$$

that can be satisfied if all eigenvalues of $\boldsymbol{\Sigma}'$ are zeros.

Another way to look at this property of $\boldsymbol{\Sigma}'$ is though the perturbative expansion. We have an equation with temporal derivative of linear operator in the LHS, and nonlinear operator in the RHS.

$$\frac{\partial}{\partial t}\nabla^2\phi = -\nabla \cdot \boldsymbol{\Sigma} \cdot \nabla\phi. \quad \text{(A38)}$$

We know the eigen solution $\{\phi_k, k = 1\ldots\infty\}$ of the linear operator in the LHS, but the exact functional form of the nonlinear operator is unknown. Multiplying by $\phi d\mathscr{V}$ and integrating by part in the entire domain $\mathscr{V}$ (of course we assume the homogeneous bc), we get

$$\frac{1}{2}\frac{\partial}{\partial t}\int\limits_{\mathscr{V}} |\nabla\phi|^2 d\mathscr{V} = -\int\limits_{\mathscr{V}} \nabla\phi \cdot \boldsymbol{\Sigma} \cdot \nabla\phi d\mathscr{V}. \quad \text{(A39)}$$

A standard procedure, if the exact form of the nonlinear dependence is unknown, is to expand the nonlinear operator in (perturbation, Taylor-like, asymptotic, whatever) series, something like this $\boldsymbol{\Sigma} = \varepsilon^0\boldsymbol{\Sigma}^0 + \varepsilon^1\boldsymbol{\Sigma}' + \varepsilon^2\boldsymbol{\Sigma}'' + \ldots$, where $\boldsymbol{\Sigma}^0$ does not depend on the amplitude of ϕ, $\boldsymbol{\Sigma}'$ depends on the amplitude linearly, $\boldsymbol{\Sigma}''$ depends on the amplitude quadratically, etc, and this dependence may be rather complicated and possibly be expressed through scalar, vector and/or tensor linear differential operators of ϕ (and ε some small parameter). But even after the expansion we know more or less accurately only the zeroth order term $\boldsymbol{\Sigma}^0$ and can obtain the solution ϕ^0 of the zeroth order system

$$\varepsilon^0 : \quad \frac{\partial}{\partial t}\nabla^2\phi^0 = -\nabla \cdot \boldsymbol{\Sigma}^0 \cdot \nabla\phi^0. \quad \text{(A40)}$$

The exact form of the higher order terms (including $\boldsymbol{\Sigma}'$) is still unknown. We only know that it should be linear on the amplitude of ϕ. What we are trying to do here is to guess the appropriate form. To do this we assume that there is a small amplitude expansion of the the full solution ϕ, such that $\phi = \varepsilon^0\phi^0 + \varepsilon^1\phi' + \varepsilon^2\phi'' + \ldots$, where ϕ', ϕ'', etc., are linear combinations of the eigen-functions ϕ_k with time

varying amplitudes.

$$\phi' = \sum_{k=1}^{\infty} a_k(t)\phi_k. \tag{A41}$$

Then we have

$$\varepsilon^1 : \quad \frac{\partial}{\partial t}\nabla^2\phi' = -\nabla\cdot\mathbf{\Sigma}^0\cdot\nabla\phi' - \nabla\cdot\mathbf{\Sigma}'\cdot\nabla\phi^0, \tag{A42}$$

$$\varepsilon^2 : \quad \frac{\partial}{\partial t}\nabla^2\phi'' = -\nabla\cdot\mathbf{\Sigma}^0\cdot\nabla\phi'' - \nabla\cdot\mathbf{\Sigma}'\cdot\nabla\phi' - \nabla\cdot\mathbf{\Sigma}''\cdot\nabla\phi^0, \tag{A43}$$

$$\dots$$

We would like to terminate our expansion at some order, we may request, for example, that ϕ'' (and all higher order terms) are zeros. But then we need to deal with the term $\nabla\cdot\mathbf{\Sigma}'\cdot\nabla\phi'$ from the last equation. We can either say that ϕ' is very small, and simply discard it, or we can try to do a little better, and use it to constrain the form of the $\mathbf{\Sigma}'$.

Multiplying by $\phi' d\mathscr{V}$ and integrating by part in the entire domain $\mathscr{V}$ (of course we assume the homogeneous bc), we get

$$\frac{1}{2}\frac{\partial}{\partial t}\int_{\mathscr{V}} |\nabla\phi'|^2 d\mathscr{V} = -\int_{\mathscr{V}} \nabla\phi'\cdot\mathbf{\Sigma}^0\cdot\nabla\phi' d\mathscr{V} - \int_{\mathscr{V}} \nabla\phi'\cdot\mathbf{\Sigma}'\cdot\nabla\phi^0 d\mathscr{V}, \tag{A44}$$

$$0 = \int_{\mathscr{V}} \nabla\phi'\cdot\mathbf{\Sigma}'\cdot\nabla\phi' d\mathscr{V}, \tag{A45}$$

where the condition (A45) should be satisfied for any arbitrary mixture of eigen-modes ϕ_k that constitute ϕ'.

Taking for example ϕ' as an arbitrary eigenmode of the axonal two dimensional cylindrical (r, z) coordinate system, i.e., $\phi' \sim \phi_\kappa(r)\phi_k(z)$, and assuming that $\mathbf{\Sigma}'(\Psi[\phi_\kappa(r)\phi_k(z)])$ has the form (17) or

$$\mathbf{\Sigma}'(\Psi[\phi_\kappa(r)\phi_k(z)]) = \Sigma' \begin{pmatrix} s_\perp xy & -s_\parallel x^2 \\ s_\parallel y^2 & -s_\perp xy \end{pmatrix} \Psi[\phi_\kappa(r)\phi_k(z)] \tag{A46}$$

with $s_\perp = s_\parallel = 1$, $x = \kappa$ and $y = k$, the expression (A37) becomes

$$\int_{\mathscr{V}} \begin{pmatrix} \kappa \\ k \end{pmatrix}^t \cdot \mathbf{\Sigma}' \cdot \begin{pmatrix} \kappa \\ k \end{pmatrix} \phi_\kappa{}^2 \phi_k^2 r dr dz = \Sigma' \begin{pmatrix} \kappa \\ k \end{pmatrix}^t \cdot \begin{pmatrix} \kappa k & -\kappa^2 \\ k^2 & -\kappa k \end{pmatrix} \cdot \begin{pmatrix} \kappa \\ k \end{pmatrix} \int_{\mathscr{V}} \Psi \phi_\kappa{}^2 \phi_k^2 r dr dz = 0. \tag{A47}$$

Based on the expression (A46), that was written for a single eigen-mode (κ, k), we can easily write an expression for $\mathbf{\Sigma}'$ in a general functional/differential form as

$$\mathbf{\Sigma}' = \Sigma' \begin{pmatrix} \frac{\partial\phi'}{\partial r}\frac{\partial\phi'}{\partial z} & \frac{\partial\phi'}{\partial z}\frac{\partial\phi'}{\partial z} \\ -\frac{\partial\phi'}{\partial r}\frac{\partial\phi'}{\partial r} & -\frac{\partial\phi'}{\partial r}\frac{\partial\phi'}{\partial z} \end{pmatrix} \frac{\Psi[\phi']}{|\nabla\phi'|^2} \tag{A48}$$

It can be easily verified that this expression is linear in ϕ' amplitude and when ϕ' is taken as the (κ, k) eigen-mode this expression is equivalent to the expression used in (A46) (transposed, as we would like the left multiplication to be orthogonal, but it does not matter in general). With this expression our condition (A45) evaluates to zero for any arbitrary ϕ':

$$\int\limits_{\mathscr{V}} \nabla\phi' \cdot \mathbf{\Sigma}' \cdot \nabla\phi' d\mathscr{V} = \Sigma' \int\limits_{\mathscr{V}} \frac{\Psi[\phi']}{|\nabla\phi'|^2} \begin{pmatrix} \frac{\partial\phi'}{\partial r} \\ \frac{\partial\phi'}{\partial z} \end{pmatrix}^t \cdot \begin{pmatrix} \frac{\partial\phi'}{\partial r}\frac{\partial\phi'}{\partial z} & \frac{\partial\phi'}{\partial z}\frac{\partial\phi'}{\partial z} \\ -\frac{\partial\phi'}{\partial r}\frac{\partial\phi'}{\partial r} & -\frac{\partial\phi'}{\partial r}\frac{\partial\phi'}{\partial z} \end{pmatrix} \cdot \begin{pmatrix} \frac{\partial\phi'}{\partial r} \\ \frac{\partial\phi'}{\partial z} \end{pmatrix} r dr dz = 0 \quad \text{(A49)}$$

However the term $\nabla \cdot \mathbf{\Sigma}' \cdot \nabla\phi'$ does not evaluate to zero identically and therefore is included in the analysis of our paper.

5 DISPERSION RELATION

Assuming no azimuthal dependence, i.e., ignoring azimuthal modes with $n \neq 0$ and assuming nonlinear form of conductivity tensor (14) equation (A20) can be written in two dimensional cylindrical coordinate system as

$$\frac{\partial}{\partial t}\left(\frac{1}{r}\frac{\partial}{\partial r}r\frac{\partial\phi}{\partial r} + \frac{\partial^2\phi}{\partial z^2}\right) = -\frac{\Sigma^0_{rr}}{r}\frac{\partial}{\partial r}r\frac{\partial\phi}{\partial r} - \Sigma^0_{zz}\frac{\partial^2\phi}{\partial z^2}$$
$$- \Sigma'_{rr}\frac{1}{r}\frac{\partial}{\partial r}r(\Psi - \overline{\Psi}_0)\frac{\partial\phi}{\partial r} - \Sigma'_{rz}\frac{1}{r}\frac{\partial}{\partial r}r(\Psi - \overline{\Psi}_0)\frac{\partial\phi}{\partial z} - \Sigma'_{zr}\frac{\partial}{\partial z}(\Psi - \overline{\Psi}_0)\frac{\partial\phi}{\partial r} - \Sigma'_{zz}\frac{\partial}{\partial z}(\Psi - \overline{\Psi}_0)\frac{\partial\phi}{\partial z} \quad \text{(A50)}$$

Using expression for ϕ from (12) and (18), keeping in (A50) only terms independent of z, multiplying by $r\phi_r(r)$ and integrating radial part across membrane bilayer

$$i\omega_k(\kappa^2 + k^2)\int\limits_1^{1+\bar\delta} r\phi_r^2 dr = (\kappa^2\Sigma^0_{rr} + k^2\Sigma^0_{zz})\int\limits_1^{1+\bar\delta} r\phi_r^2 dr + \Sigma'_{rr}\int\limits_1^{1+\bar\delta} r(\Psi_0 - \overline{\Psi}_0)\left[\frac{d\phi_r}{dr}\right]^2 dr$$
$$+ ik\frac{\Sigma'_{zr} - \Sigma'_{rz}}{2}\int\limits_1^{1+\bar\delta}\frac{d}{dr}\left[r(\Psi_0 - \overline{\Psi}_0)\right]\phi_r^2 dr + k^2\Sigma'_{zz}\int\limits_1^{1+\bar\delta} r(\Psi_0 - \overline{\Psi}_0)\phi_r^2 dr \quad \text{(A51)}$$

or

$$i\omega_k(\kappa^2 + k^2)C = (\kappa^2\Sigma^0_{rr} + k^2\Sigma^0_{zz})C + \kappa^2\Sigma'_{rr}C^r_\perp + ik\frac{\Sigma'_{zr} - \Sigma'_{rz}}{2}C_\parallel + k^2\Sigma'_{zz}C^z_\perp \quad \text{(A52)}$$

where it is convenient to introduce normalization constants $C_\perp$, $C_\parallel$, and C for radial eigenmodes of the linear model as

$$C = \int\limits_1^{1+\bar\delta} r\phi_r^2 dr = \frac{1}{\kappa^2}\int\limits_1^{1+\bar\delta} r\left[\frac{d\phi_r}{dr}\right]^2 dr = \bar\delta\int\limits_0^1 (1 + \bar\delta r)\phi_r^2 dr \approx \bar\delta\int\limits_0^1 \phi_r^2 dr, \quad \text{(A53)}$$

$$C_\perp^r = \frac{1}{\kappa^2}\int_1^{1+\bar\delta} r(\Psi_0 - \overline{\Psi}_0)\left[\frac{d\phi_r}{dr}\right]^2 dr, \tag{A54}$$

$$C_\perp^z = \int_1^{1+\bar\delta} r(\Psi_0 - \overline{\Psi}_0)\phi_r^2 dr, \tag{A55}$$

$$C_\parallel = \int_1^{1+\bar\delta} \frac{d}{dr}\left[r(\Psi_0 - \overline{\Psi}_0)\right]\phi_r^2 dr. \tag{A56}$$

6 NORMALIZATION CONSTANTS

6.1 Normalization constants for $\Psi[\phi] = -\nabla_r\phi$

Assuming function dependence of the conductivity tensor on the radial electric field $\Psi = d\phi/dr$, the average electric field in the membrane $\overline{\Psi}_0$ is

$$\overline{\Psi}_0 = \frac{1}{\bar\delta}\int_1^{1+\bar\delta} r\Psi[\phi_0]dr = -\frac{1}{\bar\delta}\int_1^{1+\bar\delta} r\frac{d\phi_0}{dr}dr = -\frac{1}{\ln(1+\bar\delta)} \tag{A57}$$

then the normalization constants $C_\perp^r$, $C_\perp^z$, and $C_\parallel$ from (A54) to (A56) are

$$C_\perp^r = \frac{1}{\kappa^2}\int_1^{1+\bar\delta} r(\Psi_0 - \overline{\Psi}_0)\left[\frac{d\phi_r}{dr}\right]^2 dr = \frac{1}{\kappa^2}\int_1^{1+\bar\delta}\frac{r-1}{\ln(1+\bar\delta)}\left[\frac{d\phi_r}{dr}\right]^2 dr \approx \frac{\bar\delta}{2}\int_0^1 \phi_r^2 dr \approx \frac{C}{2}, \tag{A58}$$

$$C_\perp^z = \int_1^{1+\bar\delta} r(\Psi_0 - \overline{\Psi}_0)\phi_r^2 dr = \int_1^{1+\bar\delta}\frac{r-1}{\ln(1+\bar\delta)}\phi_r^2 dr \approx \frac{\bar\delta}{2}\int_0^1 \phi_r^2 dr \approx \frac{C}{2}, \tag{A59}$$

$$C_\parallel = \int_1^{1+\bar\delta}\frac{d}{dr}\left[r(\Psi_0 - \overline{\Psi}_0)\right]\phi_r^2 dr = \frac{1}{\ln(1+\bar\delta)}\int_1^{1+\bar\delta}\phi_r^2 dr \approx \int_0^1 \phi_r^2 dr \approx \frac{1}{\bar\delta}C, \tag{A60}$$

hence all normalization constants for diagonal terms (C, $C_\perp^r$, $C_\perp^z$) are of the same order and the normalization constant for the off-diagonal terms ($C_\parallel$) is larger by factor $1/\bar\delta$.

6.2 Normalization constants for $\Psi[\phi] = \phi$

Assuming function dependence of the conductivity tensor on the scalar potential ϕ, the average membrane potential $\overline{\Psi}_0$ is

$$\overline{\Psi}_0 = \frac{1}{\bar\delta}\int_1^{1+\bar\delta} r\Psi[\phi_0]dr = \frac{1}{\bar\delta}\int_1^{1+\bar\delta} r\phi_0 dr = \frac{1}{\bar\delta}\int_1^{1+\bar\delta}\frac{r\ln r}{\ln(1+\bar\delta)}dr = \int_0^1 \frac{(1+\bar\delta r)\ln(1+\bar\delta r)}{\ln(1+\bar\delta)}dr \approx \frac{1}{2} + O(\bar\delta) \tag{A61}$$

then the normalization constants $C^r_\perp$, $C^z_\perp$, and $C_\parallel$ from (A54) to (A56) are

$$C^r_\perp = \frac{1}{\kappa^2}\int\limits_1^{1+\bar\delta} r(\Psi_0 - \overline{\Psi}_0)\left[\frac{d\phi_r}{dr}\right]^2 dr = \frac{1}{\kappa^2}\int\limits_1^{1+\bar\delta} r\left(\frac{\ln(r)}{\ln(1+\bar\delta)} - \frac{1}{2}\right)\left[\frac{d\phi_r}{dr}\right]^2 dr \approx \frac{\bar\delta}{6}\int\limits_0^1 \phi_r^2 dr \approx \frac{C}{6}, \quad \text{(A62)}$$

$$C^z_\perp = \int\limits_1^{1+\bar\delta} r(\Psi_0 - \overline{\Psi}_0)\phi_r^2 dr = \int\limits_1^{1+\bar\delta} r\left(\frac{\ln(r)}{\ln(1+\bar\delta)} - \frac{1}{2}\right)\phi_r^2 dr \approx \frac{\bar\delta}{6}\int\limits_0^1 \phi_r^2 dr \approx \frac{C}{6}, \quad \text{(A63)}$$

$$C_\parallel = \int\limits_1^{1+\bar\delta} \frac{d}{dr}\left[r(\Psi_0 - \overline{\Psi}_0)\right]\phi_r^2 dr = \int\limits_1^{1+\bar\delta}\left(\frac{\ln(r)}{\ln(1+\bar\delta)} - \frac{1}{2} + \frac{1}{\ln(1+\bar\delta)}\right)\phi_r^2 dr \approx \int\limits_0^1 \phi_r^2 dr \approx \frac{1}{\bar\delta}C. \quad \text{(A64)}$$

Hence again as for the case of $\Psi = -\nabla_r \phi$ all normalization constants for diagonal terms (C, $C^r_\perp$, $C^z_\perp$) are of the same order and the normalization constant for the off-diagonal terms ($C_\parallel$) is larger by factor $1/\bar\delta$.

7 NORMALIZATION CONSTANTS FOR NONLINEAR SYSTEM

It is also convenient to introduce normalization coefficient $D_\perp$, $D_\parallel$, and D for the nonlinear model that include the remaining nonlinear terms $\Psi[\phi']$ in (A50) as

$$D_\parallel = -\frac{\Sigma'_{zz}}{2C\varkappa^2}\int\limits_1^{1+\bar\delta} r\Psi[\phi_r]\phi_r^2 dr,$$

$$D_\perp = \frac{\Sigma'_{rr}}{C\varkappa^2}\int\limits_1^{1+\bar\delta} r\Psi[\phi_r]\left[\frac{d\phi_r}{dr}\right]^2 dr, \quad \text{(A65)}$$

$$D = \frac{(\Sigma'_{zr} - \Sigma'_{rz})}{4C\varkappa^2}\int\limits_1^{1+\bar\delta} \frac{d(r\Psi[\phi_r])}{dr}\phi_r^2 dr,$$

where $\varkappa^2$ is given by (26). and the normalization parameters $C_\perp$, $C_\parallel$, and C are provided in Section 5 by (A53).
